# Phylogenetic analysis of pathogenic algae reveals lineage-dependent patterns of phagocytosis

**DOI:** 10.1128/mbio.00498-25

**Published:** 2025-04-30

**Authors:** Christopher D. Shave, Mohammed J. A. Haider, Chinaemerem U. Onyishi, Megan C. McDonald, Leanne Stones, Tomasz Jagielski, Robin C. May

**Affiliations:** 1 Institute of Microbiology and Infection and School of Biosciences, College of Life and Environmental Sciences, University of Birmingham, Edgbaston, Birmingham, United Kingdom; 2 College of Medical and Dental Sciences, University of Birmingham, Edgbaston 150183 , Birmingham, England, United Kingdom; 3 Department of Biological Sciences, Faculty of Science, Kuwait University, Sabah Al-Salem University City, Kuwait City, Kuwait; 4 Molecular Mycology and Immunity Section, Laboratory of Host Immunity and Microbiome, National Institute of Allergy and Infectious Diseases (NIAID), National Institutes of Health 2511, Bethesda, Maryland, USA; 5 Department of Medical Microbiology, Institute of Microbiology, Faculty of Biology, University of Warsaw, Warszawa, Poland; The University of British Columbia, Vancouver, British Columbia, Canada

**Keywords:** *Prototheca*, phylogeny, phagocytosis, innate immunity

## Abstract

*Prototheca* is an unusual genus of algae that lack chlorophyll and are obligate heterotrophs. To date, six paraphyletic pathogenic species have been identified in the context of vertebrates, principally in cattle-associated and human-associated infections. Together with the genus *Auxenochlorella* and *Helicosporidium*, rDNA sequence analysis currently favors grouping *Prototheca* under a clade known as the Auxenochlorella, Helicosporidium and Prototheca (AHP) lineage. Most studies so far have focused only on *Prototheca bovis* and *Prototheca ciferrii* as cattle-associated species and on *Prototheca wickerhamii* as a human-associated species. However, such studies remain limited in scope as they focus on only three species of *Prototheca*, which is not representative of the total number of species within the AHP lineage. In this study, we employ a phylogenetics approach based on five new organelle-encoded genes to delineate higher-level relationships within the AHP lineage. We use the resultant data to then guide a live-cell imaging-based investigation of aspects of the mammalian innate immune response to 11 *Prototheca* species and four *Auxenochlorella* species. Our data reveal varying patterns of phagocytosis dynamics that are both host cell type- and algal species-dependent. Together, these findings reveal the interaction between pathogen phylogeny and host immune response, revealing ways to identify new therapeutic targets in the future.

**IMPORTANCE:**

Protothecosis is a rare algal infection caused by members of the genus *Prototheca*, which is comprised of unusual non-photosynthetic algae. Six pathogenic species have been identified so far that can cause infection in vertebrates, primarily cattle and humans. The phylogeny of this genus remains obscure and has been revised multiple times recently. However, this phylogeny has largely been based on only three species of *Prototheca*. To resolve this phylogenetic conundrum, here, we employ a phylogenetics approach based on five new organelle-encoded genes. We then use these data to perform live-cell imaging of a selected range of *Prototheca* species co-cultured with mammalian immune cells. Visualizing these phagocytic interactions in this context helps delineate both host cell-type- and species-dependent differences in phagocytic uptake, thereby providing novel insight into lineage-based differences.

## INTRODUCTION

*Prototheca* is a genus of achlorophyllic green algae. Unusually for green algae, six species (*Prototheca blaschkeae*, *Prototheca bovis*, *Prototheca ciferrii*, *Prototheca cutis*, *Prototheca miyajii*, and *Prototheca wickerhamii*) (1) have been reported as being able to infect mammalian hosts to cause disease. Of these, two species appear to be responsible for the bulk of infections: *P. wickerhamii* and *P. bovis*, predominantly infecting human and cattle hosts, respectively (2, 3).

The genus *Prototheca* is known to be paraphyletic; however, the extent of this paraphyly remains unclear (4). Historically, relationships based on rDNA and similar nutritional requirements indicated that the genera *Auxenochlorella* and *Helicosporidium* were part of the clade containing *Prototheca* (5–7). The lineage containing these three genera is known as the AHP lineage (6). Additionally, recent relationships based on a partial sequence of the mitochondrial cytochrome b (*cytb* or *cob*), which is now used to define species boundaries within *Prototheca*, include the genus *Chlorella* within this lineage (1, 8, 9). The genus *Chlorella* may itself be paraphyletic, including the genus *Micractinium* (10), which may therefore require the inclusion of *Micractinium* in the AHP lineage. Depending on the composition of the lineage, it may be referred to as the AHP (*Auxenochlorella*, *Helicosporidium*, and *Prototheca*), CHAP (*Chlorella*, *Helicosporidium*, *Auxenochlorella*, and *Prototheca*), or CHAMP (*Chlorella*, *Helicosporidium*, *Auxenochlorella*, *Micractinium*, and *Prototheca*) lineage.

Additionally, some higher-level relationships within *Prototheca* remain unclear. *Prototheca moriformis*, *Prototheca tumulicola*, and *Prototheca stagnora* are considered basal to the AHP/CHAP/CHAMP lineage in phylogenies based on *cytb* (8, 9), but not in phylogenies based on rDNA (11). *P. wickerhamii* has, at times, been suggested to be more closely related to the clade containing *P. cutis* and *P. miyajii* or the clade containing *Prototheca xanthoriae* and *Auxenochlorella* species (1).

**Fig 1:**
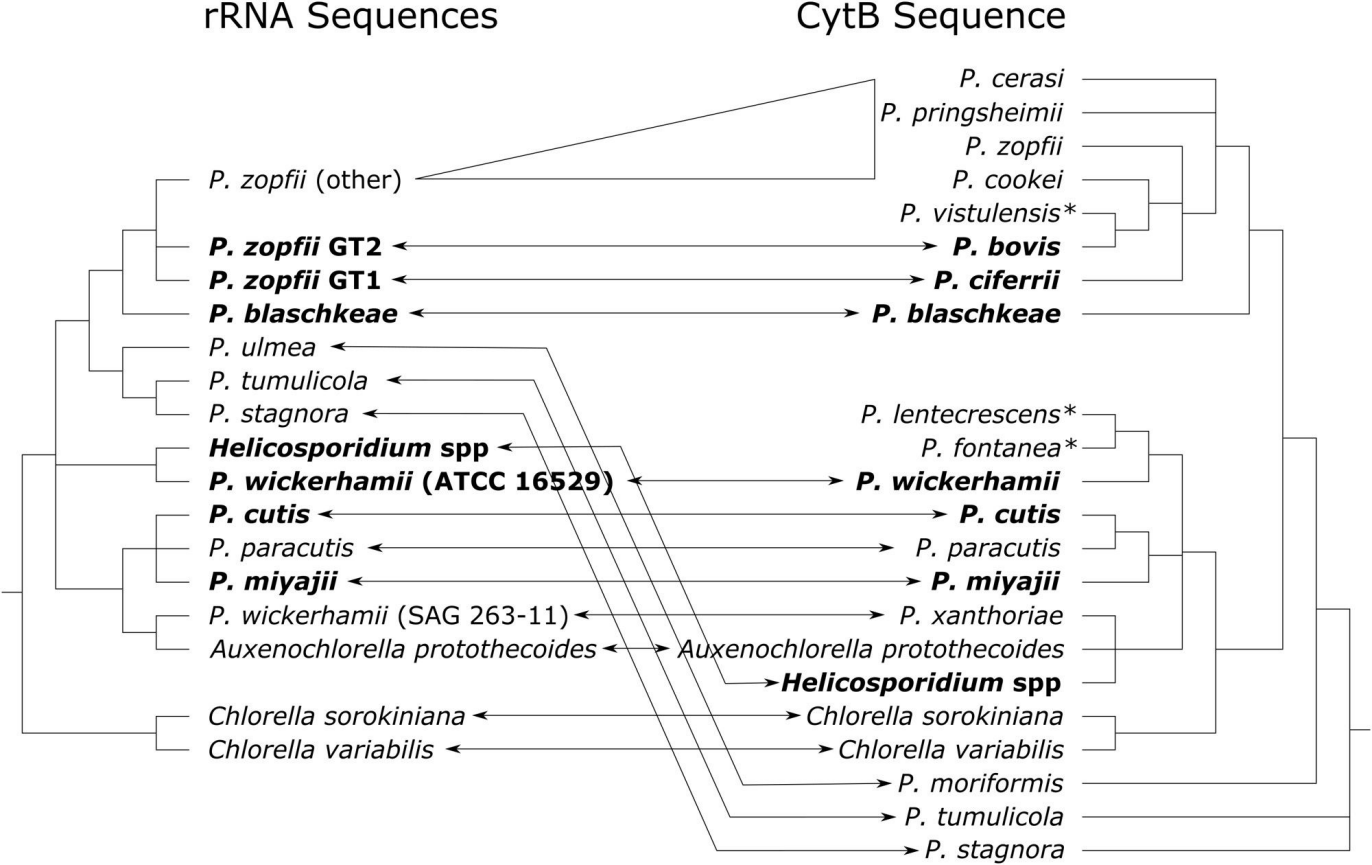
A summary of relationships between species in the lineage containing *Chlorella*, *Helicosporidium*, *Auxenochlorella*, and *Prototheca*. Two summaries are provided, reflecting conflict between relationships derived from phylogenies built using rRNA (left) or mitochondrial *cytb* (right) sequence data. All nodes shown are supported by bootstrap values of 70 or greater. Pathogenic species are highlighted in bold. Some species names and boundaries were redefined when *cytb* was chosen to define *Prototheca* species. Where possible, equivalent species classifications are indicated with arrows. Where genotype or strain information (for *Prototheca zopfii* and *P. wickerhamii*, as defined by rRNA, respectively) enables distinguishing between the former and current species, these have been provided. The expansion of former *P. zopfii* strains without a genotype into multiple new species is represented by a triangle. Species defined by *cytb* sequences without an equivalent classification defined by rRNA are indicated by asterisks. Updated from reference 4.

Pathology is not restricted to one sub-lineage within the AHP/CHAP/CHAMP lineage. Instead, pathogenic species are scattered throughout the lineage (Fig. 1). Importantly, the major pathogenic species, *P. bovis* and *P. wickerhamii*, are relatively distantly related. It has been suggested that the species in the current genus *Prototheca* could be split into at least two genera, likely separating *P. bovis* and *P. wickerhamii* (12), but it is not yet clear where new genus boundaries should be drawn or how many there should be, given the uncertainty described above.

To date, there have been few studies that compare the infectious process of different *Prototheca* species. As a result, it is not yet known if there are differences in immune interactions or pathogenic mechanisms of more distantly related *Prototheca* species, especially those for which case numbers remain low. Current research investigating pathogenic mechanisms focuses exclusively on *P. bovis*, *P. ciferrii*, and *P. wickerhamii*. It has been reported that *P. bovis* and *P. ciferrii* are able to induce pro-inflammatory responses (via mitochondrially derived reactive oxygen species or the NLRP3 inflammasome), but that *P. bovis* does so to a greater extent, and that *P. bovis* is able to kill host cells, whereas *P. ciferrii* does not (13–15). There is some evidence that *P. bovis* is able to actively invade macrophages, whereas *P. ciferrii* cannot (16). It has also been observed that *P. wickerhamii* can cause cytotoxicity against bone marrow-derived macrophages (BMDMs) (17). In a previous paper, we showed that *P. wickerhamii* is generally not phagocytosed by murine macrophages and is phagocytosed significantly less than *P. bovis* by human macrophages (18).

However, this existing work covers only three of the six known pathogenic species of *Prototheca*, which themselves are only a fraction of the total number of species within the AHP lineage. With the identification of *P. ciferrii* as a causative agent of disease, there have been no investigations into the interactions between non-pathogenic species and mammalian immunity. Thus, our understanding of the interaction between AHP algae and mammalian immunity is extremely limited in scope.

In this study, we investigate higher-level relationships in the AHP lineage using five new organelle-encoded genes and the overall arrangement of genes in whole organelle genomes. We then use these phylogenetic data to guide a live-cell imaging approach to investigate the dynamics of phagocytosis for 11 species of *Prototheca* and four species of *Auxenochlorella*. Our data show that both a murine cell line (J774A.1) and primary human macrophages phagocytose cattle-associated species of *Prototheca* (e.g*., P. bovis* and *P. ciferrii*) most efficiently compared with human-associated (e.g*., P. cutis* and *P. miyajii*) or environmental species (e.g*., P. moriformis* and *P. tumulicola*). Interestingly, a quantitative analysis of migration parameters showed no particular trend between cattle-associated and human-associated species. Thus, the clear difference in phagocytic rate between these two pathogen groups is likely to reflect a difference in aspects such as surface ligand availability, rather than a difference in inflammatory signaling.

## RESULTS

### Phagocytic behavior is different between pathogenic species

In a previous study, we reported that *P. wickerhamii* and *P. bovis* demonstrated radically different phagocytic behaviors when presented to the murine macrophage cell line J774A.1, with *P. wickerhamii* experiencing negligible phagocytosis while *P. bovis* was readily phagocytosed (18). As *P. ciferrii* was phagocytosed similarly to *P. bovis*, we initially assumed that this represented a divide between the pathogenic species: anticipating that *P. cutis* and *P. miyajii* would be phagocytosed similarly to *P. wickerhamii*, and *P. blaschkeae* would be phagocytosed similarly to *P. bovis* and *P. ciferrii*.

**Fig 2:**
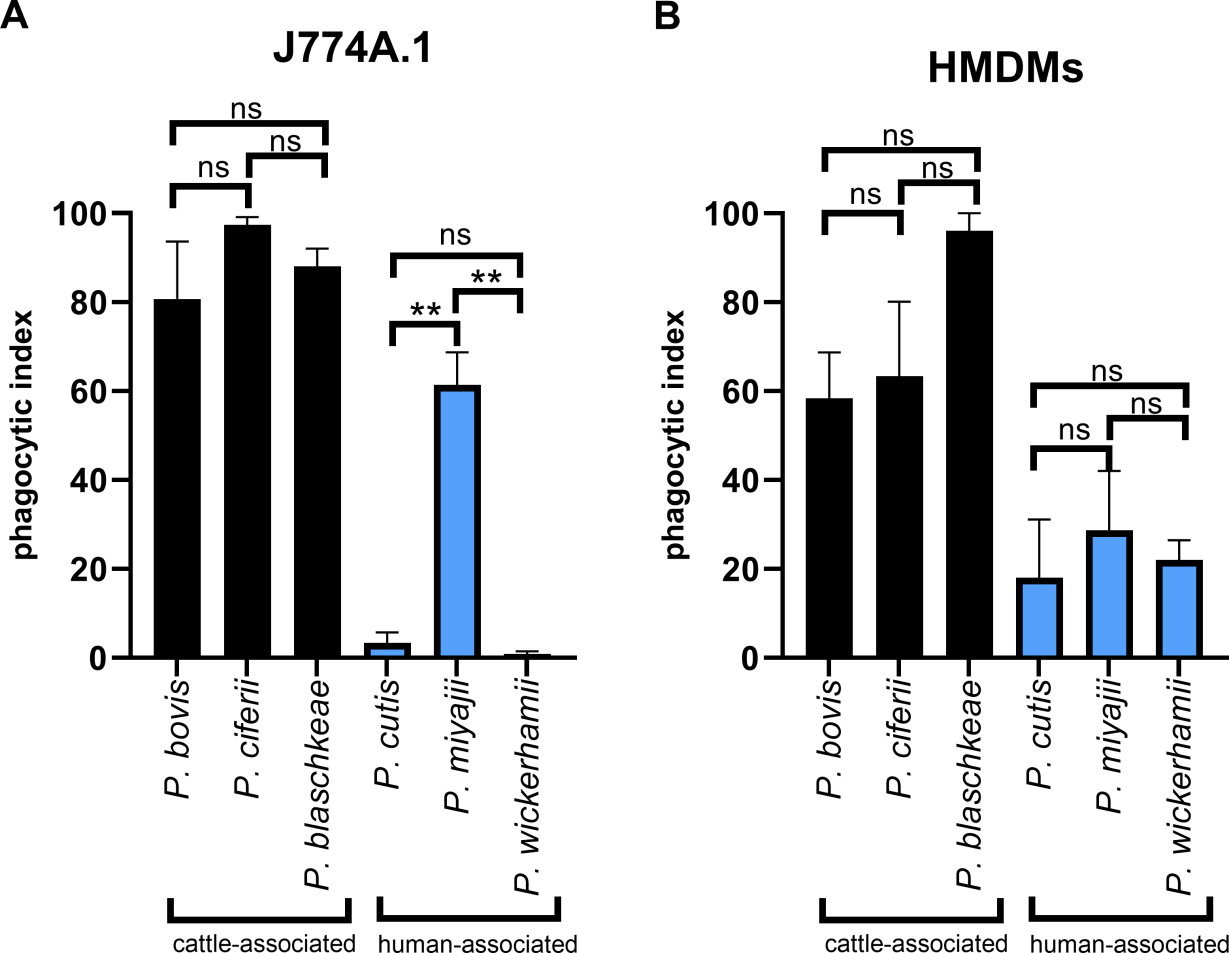
J774A.1 cells, but not HMDMs, phagocytose *P. miyajii* most efficiently compared with other pathogenic, human-associated species. J774A.1 cells and HMDMs were cultured as described in Materials and Methods, seeded in 24-well plates at 1 × 10^5^/well or 5 × 10^5^/well, respectively, and incubated at 37°C and 5% CO_2_. The next day, cells were challenged with *P. bovis* (HP3, HP40, or HP41), *P. ciferii* (HP2), *P. blaschkeae* (HP4), *P. cutis* (HP28), *P. miyajii* (HP27), or *P. wickerhamii* (HP50 or HP52) at an MOI = 3 (i.e., 3 algal cells:1 macrophage) and imaged every minute for 1 hour on either a Nikon Eclipse Ti microscope or a Zeiss Axio Observer microscope using a 20× objective lens. Data are expressed as the mean ± SEM of at least two independent, biological experiments, where *n* = at least 50 macrophages analyzed per frame of view per technical replicate per biological replicate for each strain and cell type. Videos were exported to and analyzed in Fiji for phagocytic index, defined as the percentage of macrophages that take up at least one algal cell by the end of the imaging period. (**A**) Phagocytic index for cattle-associated species exposed to J774A.1 cells. (**B**) Phagocytic index for human-associated species exposed to HMDMs. Statistical significance was assessed by an unpaired *t*-test, where *P* < 0.05 was considered significant. ns = not significant, **P < 0.01.

However, on exposing all six species to J774A.1 cells, it was readily apparent that only two of these predictions were true (Fig. 2A). The phagocytic index of *P. blaschkeae* is neither significantly different from either *P. bovis* (*P* = 0.70) or *P. ciferrii* (*P* = 0.08), nor is the phagocytic index of *P. cutis* significantly different from the phagocytic index of *P. wickerhamii* (*P* = 0.38). However, the phagocytic index of *P. miyajii* is significantly higher than that of both *P. wickerhamii* (*P* = 0.0012) and *P. cutis* (*P* = 0.0017), although not significantly different from *P. bovis* (*P* = 0.97) or *P. blaschkeae* (*P* = 0.25). *P. miyajii* therefore appears to be an interesting outlier.

Upon exposing the same species to HMDMs (Fig. 2B), the marked difference between cattle-associated and human-associated species remained, with the phagocytic index for cattle-associated species being significantly higher than for human-associated species. Interestingly, *P. miyajii* aligned with other human-related species when exposed to human phagocytes, suggesting that its previous “outlier” behavior is specific to murine cells.

Having identified unexpected variability in the phagocytic indexes of pathogenic *Prototheca*, we decided to expand the range of species to include non-pathogenic *Prototheca* and photosynthetic members of the AHP lineage. However, to be able to determine the contribution of relatedness to phagocytic behavior, as well as determine which genera should be included, it was necessary to first improve the resolution of relationships between AHP species.

### The partial sequence of *cytb* supports a monophyletic AHP clade

Previous phylogenies based on the sequence of *cytb* have identified that *Chlorella* species are within the AHP lineage. However, these phylogenies were based on a limited number of sequences from a limited number of *Chlorella* strains and exclude *Micractinium* sequences (1, 9). To resolve this, we obtained sequence data from five species of *Chlorella* and one species of *Micractinium*.

Additionally, previous phylogenies based on *cytb* use limited sequence data from *Auxenochlorella*, typically based on a single sequence from *Auxenochlorella protothecoides*. We therefore sequenced genomes from two additional species, *Auxenochlorella symbiontica* and an unknown species represented by a strain referred to here as HA7 (kindly provided by Thomas Pröschold, Universität Innsbruck, Austria). Furthermore, the genome from the algal strain FACHB-9, which was originally identified as *Chlorella pyrenoidosa*, is publicly available (19). However, *C. pyrenoidosa* has been reported as being synonymous with *Auxenochlorella pyrenoidosa* (20). Thus, this strain was considered a potential member of *Auxenochlorella*. Sequences from four *Auxenochlorella* taxa could therefore be included.

**Fig 3:**
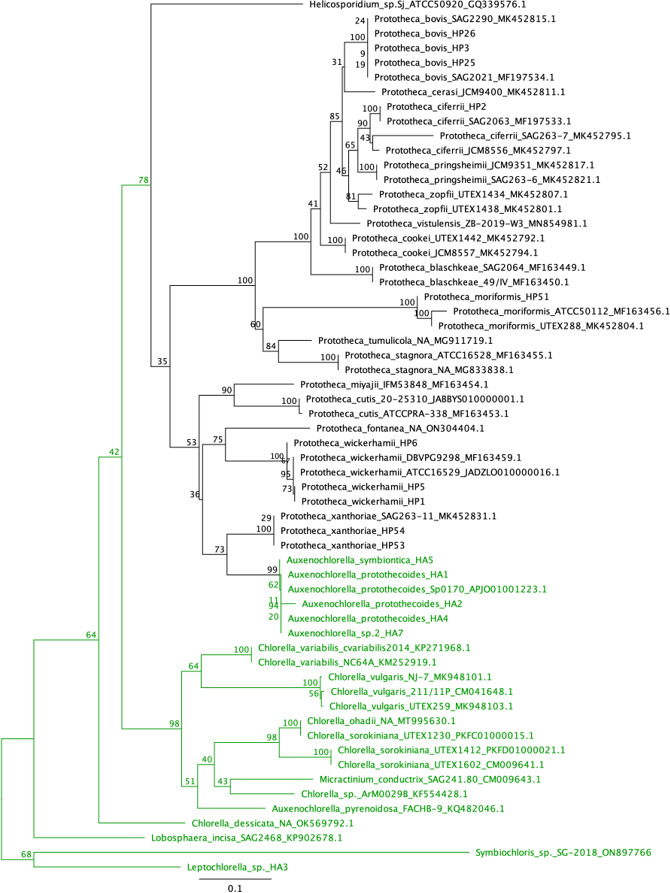
Maximum likelihood phylogeny based on partial *cytb* gene sequences of CHAMP species. This phylogeny is based on an alignment of 60 sequences of 598 nucleotides. CHAMP species are represented by the following number of genomes: *Chlorella*, 11; *Helicosporidium*, 1; *Auxenochlorella*, 7; *Micractinium*, 1; and *Prototheca*, 37. Sequences are provided with genus and species names, strain ID (if possible), and GenBank accession number (if available), separated by underscores. Numerical values at nodes represent ML bootstrap support. The labels and branches of photosynthetic lineages are colored green. To avoid long branch attraction, the phylogeny was rooted in *Symbiochloris* sp. SG-2018 and *Leptochlorella* sp. HA3 rather than a more distantly related *Chlamydomonas* sequence. Scale bar indicates nucleotide substitution rate.

A maximum likelihood (ML) phylogeny based on a 598 bp alignment of 60 sequences of *cytb*, including 37 *Prototheca* sequences, 11 *Chlorella* sequences, 7 *Auxenochlorella* sequences, 1 *Micractinium* sequence, and 1 *Helicosporidium* sequence, no longer identifies *Chlorella* as being within the AHP lineage (Fig. 3). Instead, *Chlorella* and *Micractinium* form a sister group to a monophyletic AHP clade.

**Fig 4:**
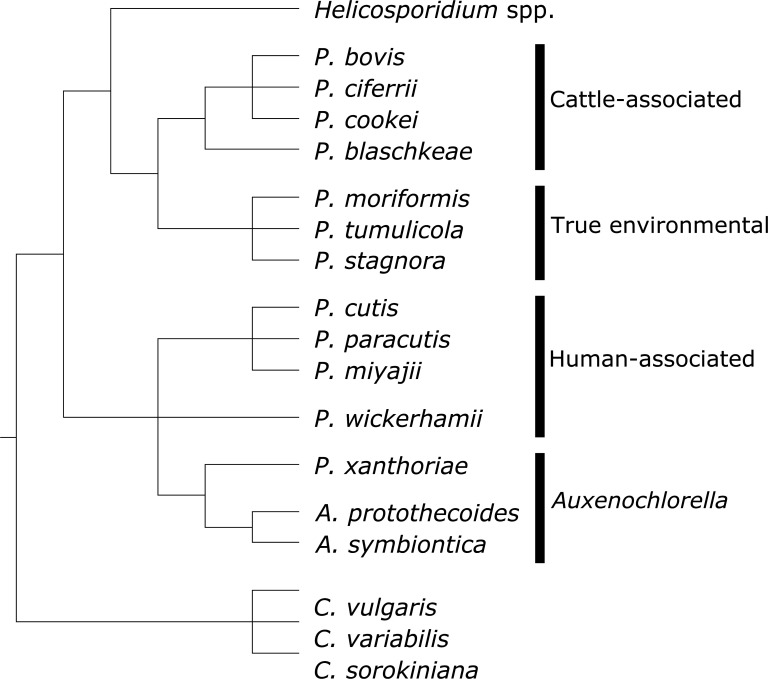
Consensus cladogram for relationships between AHP species and related taxa, based on new data. This cladogram is based on five new phylogenies based on organelle genes available in Fig. S1 and S2 and supplemented with historical rRNA phylogenies and the *cytb* phylogeny available in Fig. 3. Nodes are supported by bootstrap values of 70 in at least half of the new phylogenies, with the exception of the relationship between *P. cutis* and *P. wickerhamii,* which remains unclear. These relationships were used to allocate *Prototheca* and *Auxenochlorella* species into lineages for subsequent phagocytosis experiments.

Additionally, six well-supported groups emerge within the AHP lineage (Fig. 4). *Helicosporidium* is well supported as being basal to the lineage as a whole, forming the first group. The second group is comprised of several species that were originally designated *P. zopfii* (i.e*. P. blaschkeae*, *P. bovis*, *P. cerasi*, *P. ciferrii*, *P. cookei*, *P. pringsheimii*, and *P. zopfii*) but have been reclassified (1, 5). The third group is comprised of *P. moriformis*, *P. tumulicola*, and *P. stagnora*, which are no longer considered basal to the AHP lineage. The fourth group is comprised of species that were originally designated *P. wickerhamii* (i.e*. P. cutis*, *P. paracutis*, and *P. miyajii*) (21, 22) but notably does not include the currently defined *P. wickerhamii*, which instead forms the fifth group. The final group comprises all *Auxenochlorella* sequences and *P. xanthoriae*. It is interesting that of these six groups, only two contain no pathogenic species: groups 3 and 6.

However, some of the higher-level relationships between these groups were relatively poorly supported. We therefore investigated these relationships, using additional organelle-derived sequences.

### Other organelle sequences suggest two major subgroups within the AHP lineage and possible additional boundaries within it

Five additional genes were selected from organelle genomes: *atp1*, *cox1*, and *nad5* from mitochondrial genomes (Fig. S1) and *cysT* and *rpl2* from plastid genomes (Fig. S2). Phylogenies based on all additional genes support the separation of *Chlorella* species from the monophyletic AHP clade. *Chlorella* species were therefore not included in phagocytosis assays, as they are not members of the AHP lineage. Additionally, phylogenies based on four of five genes (all except *cox1*) support a deep split within the AHP lineage, separating groups 2 and 3 from groups 4, 5, and 6. Finally, four of five genes (all except *cox1*) associate *Helicosporidium* more closely with groups 2 and 3 than groups 4, 5, and 6.

The split between groups 2 and 3, but also their close association, is well supported in phylogenies generated from all five genes. The relationships between groups 4, 5, and 6, however, are less stable. In two phylogenies, *P. wickerhamii* is considered more closely related to *P. cutis* than *Auxenochlorella* and *P. xanthoriae*, but in two other phylogenies, there is not enough support to determine the order of relationships between these groups (i.e., bootstrap support is less than 70).

**Fig 5:**
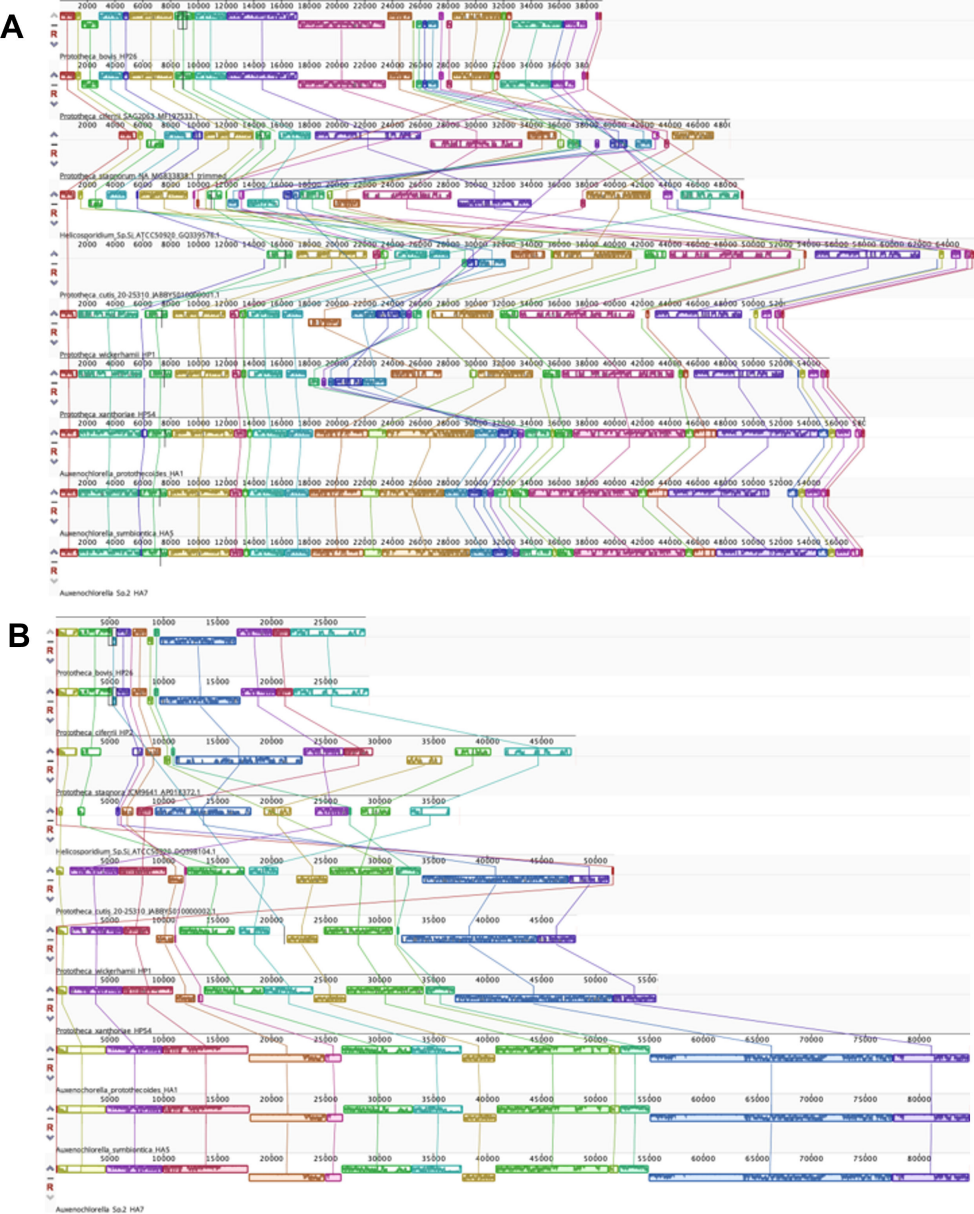
Arrangement of syntenic blocks of mitochondrial (**A**) and plastid (**B**) sequences from AHP species, as visualized in MAUVE. Circular genomes were initially aligned and oriented based on *cytb* and *CysT* sequences before iterative manual refinement. Blocks above the central line represent genes encoded on the forward strand, whereas blocks below the central line represent genes encoded on the reverse strand. Similar regions are indicated by the colors of the blocks and by connecting lines. Numbers indicate the positions along the sequence in base pairs.

To prevent over-reliance on individual gene sequences, the overall structure of organelle genomes was also investigated. Synteny supports two major subgroups, which have been reported before with a smaller range of genomes (23). There are no rearrangements between *P. bovis*, *P. ciferrii*, and *P. stagnora* mitochondrial (Fig. 5A) or plastid sequences (Fig. 5B), although *P. bovis* and *P. ciferrii* have lost a region that *P. stagnora* has retained. Similarly, there are no rearrangements between *P. wickerhamii*, *P. cutis*, *P. xanthoriae,* and *Auxenochlorella* plastid genomes, although all three *Prototheca* species have lost regions relative to the photosynthetic *Auxenochlorella* species. A single inversion separates *P. wickerhamii* and *P. cutis* mitochondrial genomes. An inversion and a translocation separate *Auxenochlorella* mitochondrial genomes from *P. wickerhamii* and *P. cutis*. The *P. xanthoriae* mitochondrial genome is structurally identical to those of all three *Auxenochlorella* species. However, these structural changes are not sufficient to distinguish lower-level groups proposed based on phylogenetic data.

### The consistency of phagocytic behavior varies across AHP sub-lineages

Based on these phylogenetic and structural data, AHP species were grouped into four related sub-lineages for phagocytosis assays. The first group, containing *P. blaschkeae*, *P. bovis*, *P. ciferrii*, and *P. cookei*, was considered to be “cattle-associated” *Prototheca* species, due to the strong association between *P. bovis* and *P. ciferrii* with cattle farm environments (4). The second group, termed “human-associated,” contained *P. cutis*, *P. miyajii*, *P. wickerhamii*, and *P. paracutis* due to their historical connection and links to human clinical protothecosis. The third group contained *P. moriformis* and *P. tumulicola*; these were considered “true environmental” *Prototheca* species, as this is the only group of the six proposed above that contains no pathogenic species while also only containing *Prototheca* species. Finally, the fourth group consisted of all *Auxenochlorella* species and *P. xanthoriae*. Importantly, this fourth group contains no pathogens but does contain a strain of *P. symbiontica* derived from an endosymbiosis with *Hydra viridis*.

**Fig 6:**
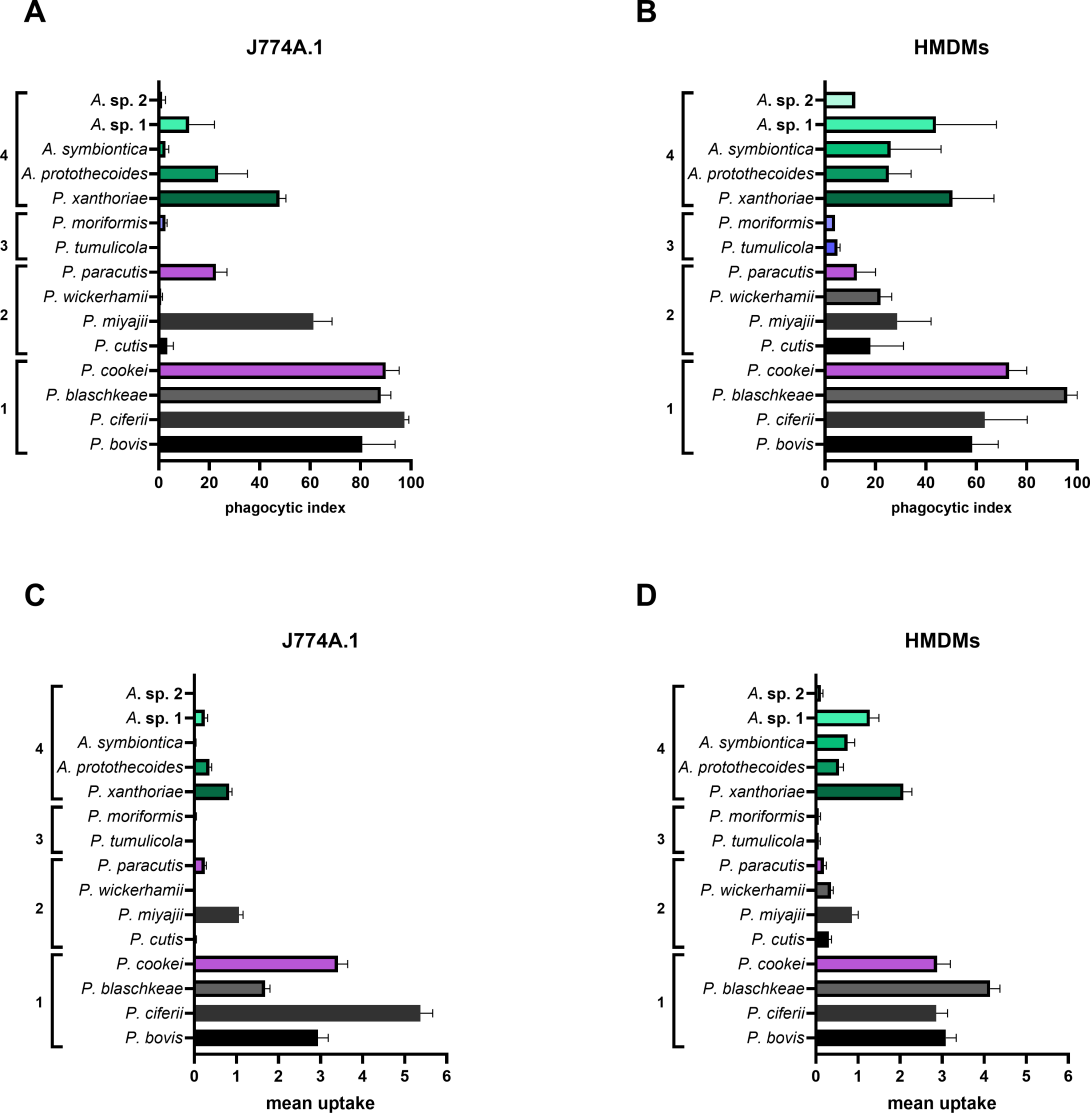
Pathogenic and non-pathogenic cattle-associated species and strains of *Prototheca* are phagocytosed most efficiently by both J774A.1 cells and HMDMs. J774A.1 cells and HMDMs were cultured as described in Materials and Methods, seeded in 24-well plates at 1 × 10^5^/well or 5 × 10^5^/well, respectively, and incubated at 37°C and 5% CO_2_. The next day, cells were challenged with *P. bovis* (HP3, HP40, or HP41), *P. ciferii* (HP2), *P. blaschkeae* (HP4), *P. cookei* (HP32), *P. cutis* (HP28), *P. miyajii* (HP27), *P. wickerhamii* (HP50 or HP52), *P. paracutis* (HP31), *P. tumulicola* (HP29), *P. moriformis* (HP51), *P. xanthoriae* (HP53 or HP54), *A. protothecoides* (HA1 or HA2), *A. symbiontica* (HA5), or one of two unidentified *Auxenochlorella* spp. (HA6 or HA7) at an MOI = 3 (i.e., 3 algal cells:1 macrophage) or MOI = 1 for some strains, and imaged every minute for one hour on either a Nikon Eclipse Ti microscope or a Zeiss Axio Observer microscope using a 20× objective lens. Data are expressed as the mean ± SEM of at least two independent, biological experiments, where *n* = at least 50 macrophages analyzed per frame of view per technical replicate per biological replicate for each strain and cell type. Videos were exported to and analyzed in Fiji for phagocytic index (**A and B**), defined as the percentage of macrophages that take up at least one algal cell by the end of the imaging period, and mean uptake (**C and D**), defined as the mean number of algal cells taken up by a single macrophage by the end of the imaging period. Statistical significance was assessed by one-way ANOVA followed by Tukey’s multiple comparisons test, where *P* < 0.05 was considered significant. Group 1: cattle-associated species. Group 2: human-associated species. Group 3: true environmental species. Group 4: *P. xanthoriae* and *Auxenochlorella* spp.

Overall, for both J774A.1 cells and HMDMs, the greatest extent of phagocytosis as assessed by both phagocytic index and mean uptake was for cattle-associated species (Fig. 6; Fig. S3 to S8). There were no statistically significant differences between any of the four species within this group, and thus, overall, it appears that this clade of cattle-associated species shares the common feature of being well recognized and engulfed by mammalian macrophages.

**Fig 7:**
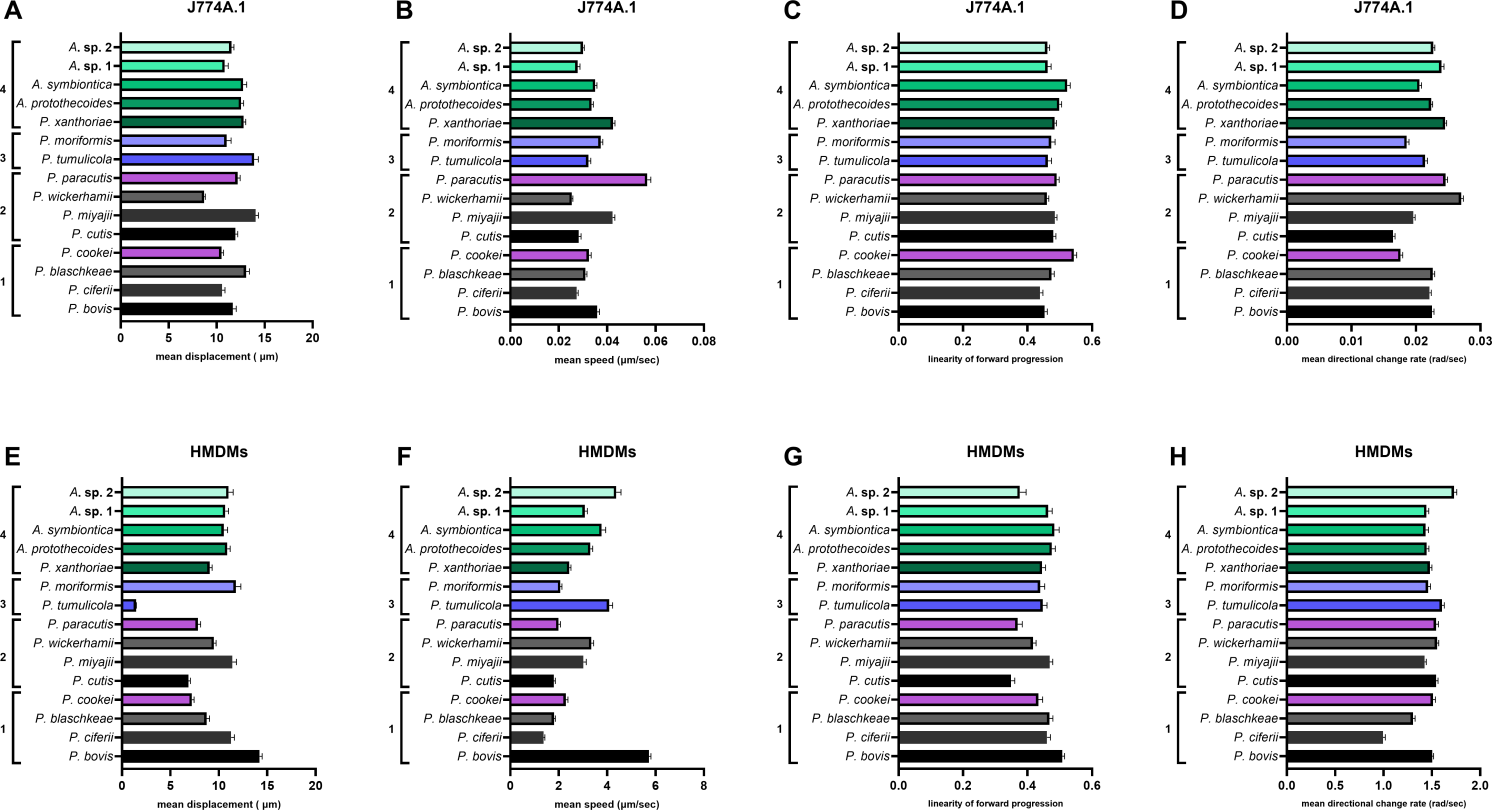
Macrophage migratory dynamics vary to different extents across sub-lineages within the AHP lineage. J774A.1 cells and HMDMs were cultured as described in Materials and Methods, seeded in 24-well plates at 1 × 10^5^/well or 5 × 10^5^/well, respectively, and incubated at 37°C and 5% CO_2_. The next day, cells were challenged with *P. bovis* (HP3, HP40, or HP41), *P. ciferii* (HP2), *P. blaschkeae* (HP4), or *P. cookei* (HP32) at an MOI = 3 (i.e., 3 algal cells:1 macrophage) and imaged every minute for 1 hour on either a Nikon Eclipse Ti microscope or a Zeiss Axio Observer microscope using a 20× objective lens. Data are expressed as the mean ± SEM of at least three independent, biological experiments, where *n* = at least 50 macrophages analyzed per frame of view per technical replicate per biological replicate for each strain and cell type. Videos were exported to and analyzed in Fiji for migratory behavior parameters. (**A and E**) Data show mean displacement (μm). (**B and F**) Data show mean speed (μm/s). (**C and G**) Data show linearity of forward progression (fraction of 1). (**D and H**) Data show mean directional change rate (rad/sec). Statistical significance was assessed by one-way ANOVA followed by Tukey’s multiple comparisons test, where *P* < 0.05 was considered significant. Group 1: cattle-associated species. Group 2: human-associated species. Group 3: true environmental species. Group 4: *P. xanthoriae* and *Auxenochlorella* spp.

Next, we used one-way ANOVA followed by Tukey’s multiple comparisons test to perform a more rigorous series of pairwise comparisons between all species across all sub-lineages for each macrophage type. We analyzed phagocytic index (Table S1) and mean uptake (Table S2). Overall, we noted some robust differences between the different groups. However, within each group, species were largely similar in the observed pattern of both metrics, with the possible exception of *P. miyajii*. Taken together, these data indicate that there are species-specific differences in phagocyte interaction which partially, but not wholly, align with wider taxonomic lineages. We note that the limited number of *Prototheca* strains currently available significantly restricts the power of any statistical analysis. Consequently, a more robust quantitative test of lineage-dependent host interactions will need to await the collection of additional strains, especially from within the underrepresented species, such as *P. miyajii* or *P. ciferrii*.

**TABLE 1:** List of species and strains used in this study

H_number	Species	Host	External names
HP1	*P. wickerhamii*	Human	ATCC 30395
HP2	*P. ciferrii*	Cattle	SAG2063
HP3	*P. bovis*	Cattle	SAG2021
HP4	*P. blaschkeae*	Human	SAG2064
HP27	*P. miyajii*	Human	IFM 53848
HP28	*P. cutis*	Human	ATCC PRA-338
HP29	*P. tumulicola*	None	JCM 31123
HP31	*P. paracutis*	None	JCM 32112; YMTW3-1; TBRC8745
HP32	*P. cookei*	None	JCM 8557
HP40	*P. bovis*	Cattle	E9
HP41	*P. bovis*	Cattle	SR4
HP50	*P. wickerhamii*	Cattle	UTEX 1437; ATCC 16523; NRRL Y-2464
HP51	*P. moriformis*	Unknown	CBS614.66
HP52	*P. wickerhamii*	Human	JCM 9645; CBS 608.66
HP53	*P. xanthoriae*	None	CBS 612.66
HP54	*P. xanthoriae*	None	JCM 9729; CBS 611.66
HA1	*Auxenochlorella protothecoides*	None	CCAP 211/8D; SAG 211–8d
HA2	*Auxenochlorella protothecoides*	None	CCAP 211/7A
HA3	*Leptochlorella* sp.	None	CCAP 211/54
HA5	*Auxenochlorella symbiontica*	*Hydra viridis*	CCAP 211/61
HA6^*a*^	*Auxenochlorella* sp.1	Unknown	Unknown
HA7^*a*^	*Auxenochlorella* sp.2	Unknown	Unknown

^*a*^ Strains HA6 and HA7 were kindly provided by Dr. Thomas Pröschold. They have not yet been characterized to species level or provided with strain identifiers.

Overall, we observed greatly reduced (or absent) phagocytosis for some of the human-associated and true environmental species. However, despite the fact that macrophages do not appear to recognize these species for engulfment, live-cell imaging videos nonetheless reveal active migration of macrophages toward all human-associated *Prototheca* species (Movies S1 and S2). To examine this in greater detail, we performed a similar series of pairwise comparisons using one-way ANOVA followed by Tukey’s multiple comparisons test. We assessed four metrics that describe macrophage migratory dynamics as explained in Materials and Methods. Specifically, this included an assessment of macrophage mean displacement, mean speed, linearity of forward progression, and mean directional change rate (Fig. 7). For all of the metrics assessed, there were no major differences in migration dynamics between species (Tables S3 to S6). Thus, macrophages recognize and migrate toward all of these pathogens in an equivalent manner, although their subsequent binding and engulfment are highly species variable

## DISCUSSION

This study represents a combined investigation into the phylogenetic relationships and phagocytic behavior of the AHP lineage. To the best of our knowledge, it is the first investigation into interactions between *Prototheca* and the mammalian immune system that spans the genus, rather than focusing on one or two species. Moreover, this is the first study to include additional sequences from *Auxenchlorella* and *Chlorella* species. Together, these threads of investigation have identified sub-lineages of *Prototheca* within the AHP lineage and revealed a surprising degree of complexity to the interactions between these sub-lineages and mammalian immunity.

First, there are clear differences between the recognition of different *Prototheca* species by murine macrophages. This suggests that there are differences between different *Prototheca* species that are relevant for their interaction with some mammalian immune systems. This may present one basis for host preference. One possibility is that different species have different pathogen-associated molecular patterns (PAMPs) on their surfaces, either on their plasma membrane or as components of their cell wall, only some of which can be recognized by macrophage pattern recognition receptors (PRRs) for phagocytosis. This has previously been demonstrated for five pathogenic species of the fungal genus *Candida*, where it was observed that differences in cell wall composition influenced differential uptake by human macrophages and the consequent innate immune response (24). It is therefore plausible that species-specific variations in PAMPs would likely account for the phagocytic index of *P. cookei* resembling that of its pathogenic close relatives *P. bovis* and *P. ciferrii*. If so, it is unclear what these PAMPs are or whether they are the same in all phagocytosed *Prototheca* species. For example, it seems unlikely that *P. bovis*, *P. miyajii*, and *P. xanthoriae*, all of which are phagocytosed readily but seem to be distantly related, are recognized by the same surface components, when closer relatives like *P. paracutis* and some *Auxenochlorella* species are phagocytosed at much lower levels. Early studies have suggested the involvement of β-(1,4)glucan (25); glucose, galactose, mannose, and hexosamine-based cell wall structures (26); as well as sporopollenin in recognition by phagocyte PRRs (27). Nonetheless, the engagement of these putative PAMPs with specific sets of host cell receptors remains to be conclusively proven.

A second possibility is that some *Prototheca* species actively invade macrophages rather than being passively phagocytosed. This may account for the elevated phagocytic index of *P. miyajii* compared with its close relatives *P. paracutis* and *P. cutis*. However, invasion is unlikely to be sufficient to account for all of the differences observed in phagocytic indexes across *Prototheca* species. It has already been shown that *P. bovis* is able to invade mammalian cells, whereas *P. ciferrii* is not (28), and *P. bovis* was not phagocytosed more than *P. ciferrii* using any metric in either cell type. Currently, only *P. bovis* has been suggested to actively invade mammalian cells. Although it is plausible, future work should address this possibility more conclusively.

A third possibility is that some *Prototheca* species actively inhibit phagocytosis. This could account for the very low phagocytic indexes of *P. wickerhamii* and *P. cutis* compared with *P. paracutis* and some *Auxenochlorella* species. The true environmental species, *P. tumulicola* and *P. moriformis*, have unusually low phagocytic indexes in human macrophages as well as in murine macrophages.

Importantly, however, these possibilities are not mutually exclusive, and it is possible that all three are relevant in accounting for these trends of phagocytosis across the AHP lineage. Future work should elucidate the mechanisms of phagocytosis of *Prototheca*. At least some of this difference is likely to originate from non-infectious selection pressures, as *P. xanthoriae* appears to have different phagocytic behaviors to *Auxenochlorella* despite being the closest *Prototheca* relative to *Auxenochlorella*, and all species are non-pathogenic. These phenotypic data, combined with the sub-lineages identified from organelle gene sequences, may also support the separation of *Prototheca* into up to five new genera.

It is interesting that there appear to be different trends in phagocytosis between cells derived from different host species. Several strains (i.e*., P. cutis*, *P. wickerhamii*, and *A. symbiontica*) appear to have extremely low or negligible levels of phagocytosis in J774A.1 cells but are phagocytosed more readily by HMDMs. This suggests some strains are recognized by some macrophages but not by others. The most likely explanation for this is that different *Prototheca* species may have differing profiles of PAMPs on their surface, thereby altering their recognition by host phagocytes. Varying PAMP levels are known to occur in other eukaryotic pathogens such as the fungus *Candida albicans* (29)*,* and therefore, it seems plausible that similar differences may occur in *Prototheca*. To date, however, no specific PAMPs have been identified in this group of pathogens—once they are, it will be of great interest to test whether they are differentially displayed between strains in a way that may explain the phagocytic variability that we have identified. Differences between murine and human immune responses to *Prototheca* have been observed previously (16), potentially reflecting differences in the pathogen recognition receptors deployed by mouse versus human phagocytes.

Finally, the work presented here does not rule out additional variability in the phagocytic dynamics of the AHP lineage or in other interactions between the AHP lineage and mammalian immunity. Additionally, different strains of *P. wickerhamii* have been observed to cause different levels of cytotoxicity in BMDMs (17), and different strains of *P. ciferrii* have been reported to induce different cytokine responses in J774A.1 cells (30). Only a limited repertoire of strains was explored here, with most species only represented by one strain, and hence, it may be that additional complexity remains within the phagocytic dynamics of each *Prototheca* species.

## MATERIALS AND METHODS

All chemicals were sourced from Sigma Aldrich, unless specified otherwise.

### Strains

Strains were acquired from a variety of collaborators, and AHP strains were assigned internal strain identifiers summarised in Table 1. *Prototheca* strains HP1 to HP26 were a kind gift from Uwe Rösler (Free Universität, Berlin). *Auxenochlorella* strains HA1 to HA5 were purchased from the Culture Collection of Algae and Protozoa (CCAP). *Auxenochlorella* strains HA6 and HA7 were a kind gift from Thomas Pröschold (Universität Innsbruck, Austria).

### Algal cultures

Working stocks of *Prototheca* and *Auxenochlorella* isolates were normally maintained on *Prototheca* isolation medium (PIM) agar without 5-fluorocytosine (7), grown at 25°C. Briefly, PIM agar was made by dissolving 10 g potassium hydrogen phthalate (C_8_H_5_KO_4_), 0.9 g sodium hydroxide (NaOH), 0.1 g magnesium sulfate (MgSO_4_), 0.2 g potassium hydrogen phosphate (KH_2_PO_4_), 0.3 g ammonium chloride (NH_4_Cl), 10 g glucose (C_6_H_12_O_6_), 1 mg thiamine hydrochloride (C_12_H_18_Cl_2_N_4_OS), and 20 g agar in 1 L distilled water and autoclaving at 121°C for 15 min. Colonies were used to inoculate liquid cultures grown in Sabouraud dextrose broth, grown at 25°C for 48 h, which provided material for DNA extraction and infection assays described below.

Due to slow growth and a seemingly greater reliance on light, cultures of HA6 were grown on PIM agar under natural sunlight at approximately 20°C. Cultures reliably took 10+ days to grow. Cells were resuspended in Dulbecco’s PBS for infection.

### DNA extraction and sequencing

As part of a project to sequence the genomes of AHP species, DNA was extracted from 11 strains of *Prototheca* and three strains of *Auxenochlorella*.

To extract DNA from cultures, cells were pelleted by centrifugation at 5,000 *g* for 1 min, resuspended in 20 mM NaOH, and flash frozen in liquid nitrogen. Once thawed, an equal volume of 0.5 mm glass beads was added. The pellet and glass beads were resuspended in 400 µL of CTAB lysis buffer (2% cetyltrimethylammonium bromide [CTAB], 1.4 M NaCl, 100 mM Tris base [pH 8.0], and 20 mM ethylenediaminetetraacetic acid [EDTA]), to which 400 µL of phenol:chloroform:isoamyl alcohol (25:24:1) was added. Tubes were vortexed four times for 40 s (160 s total) at high speed to lyse cells (31). DNA was precipitated with 100% room-temperature isopropanol, washed with 70% ethanol, air dried, and resuspended in EB buffer (10 mM Tris-Cl, pH 8.5).

The length of the extracted DNA was confirmed by agarose gel electrophoresis in a 0.75% Tris base, acetic acid, and EDTA (TAE) gel run for 60 min at 70 V. To assess purity, 260/230 and 260/280 ratios were measured on a Nanodrop 2000c spectrophotometer (ThermoFisher), and concentration was quantified using a Qubit3 fluorometer and the Qubit dsDNA high sensitivity kit (ThermoFisher). Paired-end Illumina sequencing was provided by MicrobesNG (https://www.microbesng.com; standard service).

### Genome assembly

Genomes were assembled using SPAdes (v3.14.1) with default settings (32). This was facilitated by the University of Birmingham’s BlueBEAR HPC service, which provides a High Performance Computing service to the University’s research community. See http://www.birmingham.ac.uk/bear for more details. Assemblies were highly fragmented, as indicated by QUAST (v5.0.2) (33) and BUSCO (v5.3.1) (34). However, these assemblies contained contigs approximately the length of published organelle genomes.

Contigs representing complete organelle genomes were identified with BLAST (v2.12.0) (35–37) using published *cytb* sequences (MF163459 and MZ604428.1) and sequences taken from published plastid genomes (NC_054192, bases 1000–2000; MF197536.1, bases 1000–1700; KJ001761.1, bases 1000–2000) and were extracted from the overall assemblies using Samtools (v1.9, using htslib v1.9) (38). The presence of other organelle-specific genes was confirmed using GeSeq (v2.03) (39).

### Phylogenetics

In addition to the sequence data generated as described above, publicly available data were taken from GenBank. To investigate the phylogenetic placement of the CHAMP lineage, genomes and genes were taken from species within *Chlorella*, *Helicosporidium*, *Auxenochlorella*, *Micractinium*, and *Prototheca. Chlamydomonas* is often chosen as an outgroup for phylogenetics in the AHP/CHAP/CHAMP lineage. However, *Chlamydomonas* is in a different class to *Prototheca*, making them very distantly related and potentially increasing the risk of long-branch attraction. Here, sequences from *Lobosphaera* and *Symbiochloris* were chosen as outgroups to reduce this risk.

Five genes, present in all organelle genomes, were selected for phylogenetics in addition to the mitochondrial encoded gene, *cytb*. Whole organelle genomes were chosen in preference over nuclear genomes because they are easier to assemble from short-read data and therefore more easily expanded in later studies. Furthermore, the choice of *cytb* was informed by an earlier study (40) in that it was the only one among six candidate genes that could be amplified by a single set of primers. Importantly, they are all known to be involved in respiratory functions, and it is unlikely that they have undergone significant evolutionary change despite variations in the capabilities of different AHP species. Overall, all chosen genes are present in all AHP species, are of an appreciable length (i.e., over 900 bp), and are well-conserved despite the diversity of ecological niches of different AHP species.

To generate phylogenies based on individual genes, sequences were identified either with BLASTn, using sequences from AHP species as queries, or using the annotations produced by GeSeq.

In identifying sequences, some genes showed matches that were disrupted by inserts. For example, all available genomes for *Chlorella vulgaris* show the same 1350 bp insert in the sequence for *cytb*. Where inserts were found, they were manually removed before aligning to prevent interference with the alignment algorithm.

Sequences were aligned using Clustal Omega (v1.2.3) (41) in Geneious 2023.1.2 (https://www.geneious.com) with the default settings, unless specified otherwise. Phylogenetic trees were generated using the RAxML plugin (v8.2.11) (42) in Geneious, using a generalized-time reversible (GTR) GAMMA substitution model and 500 bootstrap replicates.

The arrangement of regions within organelle genomes was also explored to investigate relationships within the CHAMP lineage. To show meaningful structural differences, organelle genome sequences must be in the same orientation and start in the same place, as organelle genomes are circular. This was done manually, initially using sequences of *cytb* and *cysT* for mitochondrial and plastid genomes, respectively, followed by manual refinement. Syntenic regions were visualized using the MAUVE (v1.1.3) (43) plugin in Geneious.

### Tissue culture

To determine whether the sub-lineages identified might have different interactions with mammalian immunity, algal cells were used to infect J774A.1 murine macrophages (the European Collection of Authenticated Cell Cultures) or human monocyte-derived macrophages (HMDMs). J774A.1 cells were grown in Dulbecco’s modified Eagle’s medium with low glucose, supplemented with 10% live fetal bovine serum, 2 mM L-glutamine, and 100 U/mL penicillin-streptomycin. Prior to infection, cells were seeded in 24-well plates at 1 × 10^5^ cells per well and were activated in an equivalent medium without serum and with 150 ng/mL phorbol 12-myristate 13-acetate (PMA) for at least 1 h.

HMDMs from two donors were prepared as described previously (18). Prior to infection, human cells were seeded in 24-well plates at 5 × 10^5^ cells per well in a complete RPMI 1640 medium supplemented with 10% heat-inactivated human serum and 20 ng/mL human GM-CSF (PeproTech, London, UK) for macrophage differentiation. Cells were incubated for 6 days, with a medium replacement on day 3. Cell lines and primary cells were routinely incubated at 37°C and 5% CO_2_.

### Phagocytosis assays and live-cell imaging

Algal cells were washed two or three times, depending on the number of recoverable cells, in Dulbecco’s PBS. To pellet, cells were spun at 2,700 × *g* at 20°C for 1 min. Pellets were resuspended by pipetting rather than vortexing, as *P. blaschkeae, P. bovis, P. ciferrii,* and *P. cookei* demonstrated a propensity to stick to the plastic rather than stay in suspension due to their hydrophobicity. Cells were counted on a CytoSmart automated cell counter (CytoSMART Technologies, Corning, NJ, USA).

Macrophages were infected at a multiplicity of infection (MOI) of 3:1 (i.e., three algal cells per macrophage) for most algal species. Due to difficulties in counting strains related to *P. bovis*, which often resulted in undercounting algal cell cultures used to infect macrophages, in later experiments with these strains, macrophages were infected at an MOI of 1:1.

Phagocytosis with J774A.1 cells was done in CO_2_ independent medium (ThermoFisher Scientific), whereas phagocytosis with HMDMs was done in a complete RPMI 1640 medium supplemented with 10% heat-inactivated human serum. For all assays, cells were stained with LysoTracker Red DND-99 (LTR) (ThermoFisher Scientific) at 100 nM as per the manufacturer’s instructions.

Time lapse images were taken on either a Nikon Eclipse Ti microscope or a Zeiss Axio Observe microscope fitted with an environmental control chamber set at 37°C and 5% CO_2_. Images were acquired using a 20× objective for 1 h. Images were taken at variable intervals across different experiments, ranging from 30 to 60 s. Videos were analyzed in Fiji (ImageJ).

### Analysis of live-cell imaging videos

Live imaging quantification was performed using Fiji based on a previously adopted methodology (44). At least 50 macrophages per field of view per technical replicate per species/strain per biological replicate were analyzed. Cells were scored manually by eye for uptake, and the phagocytic index of a given replicate was calculated as the percentage of macrophages that had phagocytosed at least one algal cell by the end of the imaging period. The mean uptake was calculated as the mean number of algal cells phagocytosed by a single macrophage by the end of the imaging period.

To study macrophage migratory dynamics, four metrics were analyzed using the TrackMate plugin (v7) in Fiji (45):

- Mean displacement (the mean distance—in micrometers—between a macrophage’s initial and final XY positions)
- Mean speed (the mean speed—in micrometers/second—of a macrophage).
- Linearity of forward progression (an indication of directionality, where numbers approaching a value of 1 signified overall directed motion)
- Mean directional change rate (the rate—in radians/second—at which a macrophage changes its direction on XY coordinates)

Video files (.nd2 or .czi format) were opened in Fiji, adjusted for scale, brightness, and contrast, and then saved as .tiff files. The differences in Gaussian (DoG) detector were then used, and the estimated object diameter was set to 30–40 µm depending on macrophage type. Subpixel localization was enabled, and a preview image was generated. After confirming object identification, the detection was run at 12 frames simultaneously and 1 thread per frame. Initial thresholding was then set, and spots were filtered according to quality, radius, and contrast. Tracks were then generated using the simple linear assignment problem (LAP) tracker option, with the following settings: linking maximum distance (15 µm), gap-closing maximum distance (15 µm), and gap-closing maximum frame gap (2). Tracks were then filtered according to track ID, total distance traveled, and number of spots in the track. Track features, including (but not limited to) mean displacement, mean speed, linearity of forward progression, and mean directional change rate were then exported as .csv files and further analyzed in GraphPad Prism (Version 9.3.1).

Note that this approach has several limitations that should be borne in mind. First, TrackMate depends primarily on Gaussian-based blob detection, and hence, it struggles with low signal-to-noise ratio (SNR) images. Second, TrackMate works optimally with spherical or rounded objects, and performance is reduced when working with irregularly shaped objects, including macrophages. Third, frames that contain densely packed cells may cause TrackMate to mislink tracks or lose objects because of ambiguous assignments. Mislinking tracks often requires manual correction, which is time-consuming and tedious for large data sets. Furthermore, TrackMate struggles in the case of splitting or merging objects, as is often the case for macrophages undergoing mitosis or internalizing target cargo, respectively.

### Statistical analyses

Statistical analysis was done in GraphPad Prism (Version 9.3.1). At least two independent experiments were performed for each condition. For each experiment, at least 50 macrophages were analyzed per frame of view per technical replicate. Data are expressed as the mean ± SEM of at least two independent experiments. Statistical significance was assessed by one-way ANOVA followed by Tukey’s multiple comparisons test. For all experiments, *P* < 0.05 was considered significant.

## Data Availability

All genome sequence data are freely available via the Sequence Read Archive (https://www.ncbi.nlm.nih.gov/sra) via accession PRJNA1244913.

## SUPPLEMENTAL MATERIAL

Supplemental material mbio.00498-25-s0001.pdf Supplemental figures and tables.

Movie legends mbio.00498-25-s0002.docx Legends for the two supplemental movies.

Movie S1 mbio.00498-25-s0003.avi J774A.1 cells stained with LTR phagocytosing *P. cutis* (HP28).

Movie S2 mbio.00498-25-s0004.avi J774A.1 cells stained with LTR phagocytosing *P. cutis* (HP29).

ASM does not own the copyrights to Supplemental Material that may be linked to, or accessed through, an article. The authors have granted ASM a non-exclusive, world-wide license to publish the Supplemental Material files. Please contact the corresponding author directly for reuse.
