# Supplementary material for: Phylogenetic analysis of pathogenic algae reveals lineage-dependent patterns of phagocytosis
Source: mBio. 2025 Apr 30;16(6):e00498-25. doi: 10.1128/mbio.00498-25 (PMC12153285; doi:10.1128/mbio.00498-25)
Supplement: Supplemental material — Supplemental figures and tables. [file mbio.00498-25-s0001.pdf]

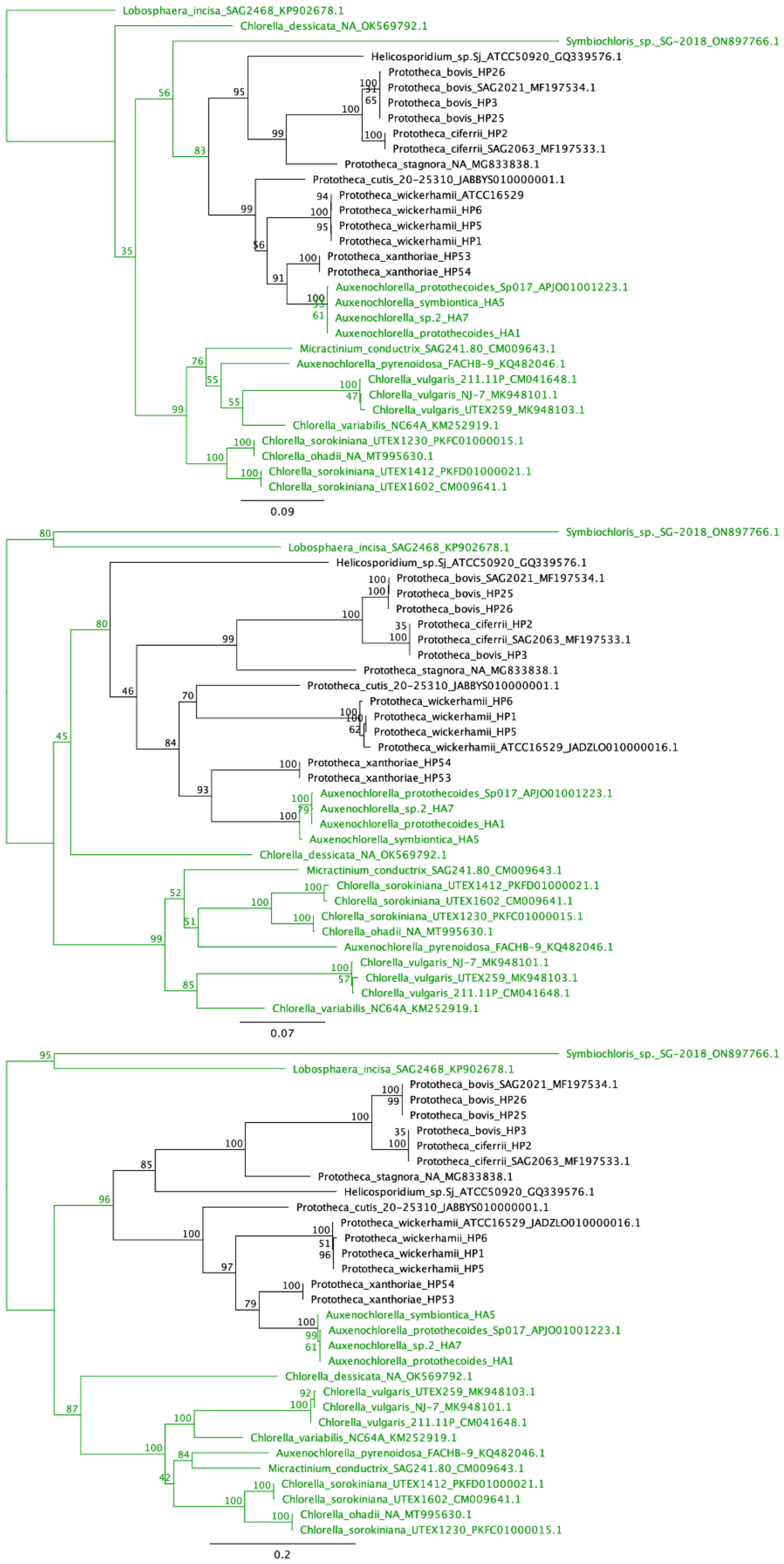

Sup. Fig. 1 Mitochondrial phylogenies. Maximum likelihood phylogenies based on mitochondrial gene sequences: *atp1* (top), *cox1* (middle), and *nad5* (bottom). These phylogenies are based on alignments of 32 sequences of 1542, 1584, and 2041 nucleotides, respectively. CHAMP species are represented by the following number of genomes: *Chlorella*, 9; *Helicosporidium*, 1; *Auxenochlorella*, 5; *Micractinium*, 1; *Prototheca*, 14. Sequences are provided with genus and species names, strain ID (if possible) and GenBank accession number (if available), separated by underscores. Numerical values at nodes represent ML bootstrap support. The labels and branches of photosynthetic lineages are coloured green. Phylogenies were rooted to *Lobosphaera incisa* SAG2468 and *Symbiochloris* sp. SG-2018. Scale bar indicates nucleotide substitution rate.

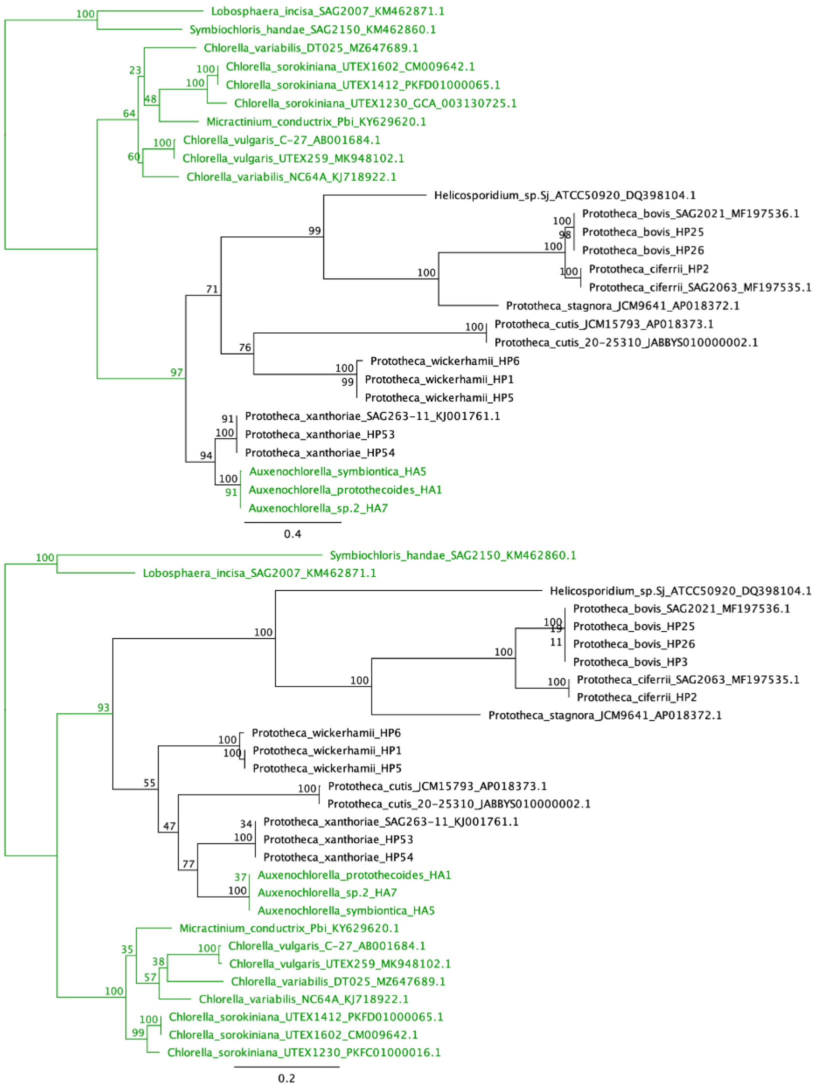

Sup. Fig. 2 Plastid phylogenies. Maximum likelihood phylogenies based on plastid gene sequences: *cysT* (top) and *rpl2* (bottom) sequences of CHAMP species. These phylogenies are based on alignments comprised of 28 and 29 sequences of 1070 and 930 nucleotides, respectively. CHAMP species are represented by the following number of genomes: *Chlorella*, 7; *Helicosporidium*, 1; *Auxenochlorella*, 3; *Micractinium*, 1; *Prototheca*, 14 or 15. Sequences are provided with genus and species names, strain ID (if possible) and GenBank accession number (if available), separated by underscores. Numerical values at nodes represent ML bootstrap support. The labels and branches of photosynthetic lineages are coloured green. Phylogenies were rooted to *Lobosphaera incisa* SAG2468 and *Symbiochloris* sp. SG-2018. Scale bar indicates nucleotide substitution rate.

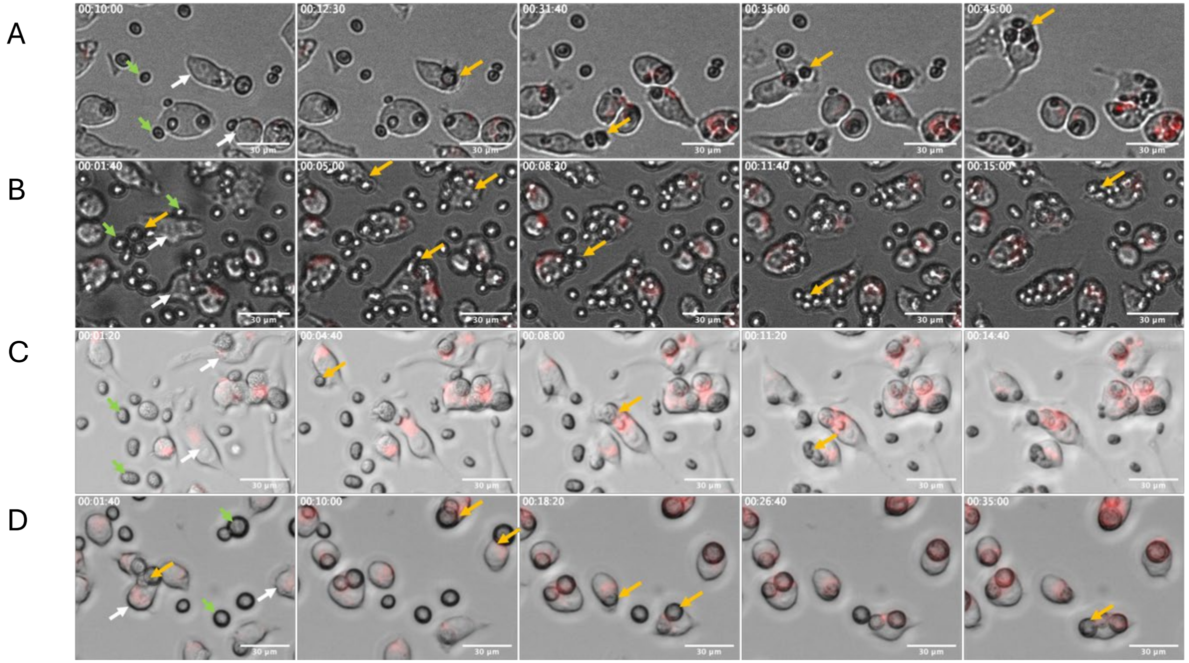

Sup. Fig. 3 Phagocytosis of cattle-associated species of *Prototheca* by J774A.1 cells. Algal cells are indicated by green arrows; macrophages are indicated by white arrows; phagocytosis events are indicated by yellow arrows. LTR staining indicates acidified compartments within the macrophages – primarily acidified phagolysosomes containing phagocytosed algal cells. Rows show selected frames from videos, at times indicated by the time stamp in the top left. The species and strain of the algal cells in each row are A) *P. bovis*, HP3; B) *P. ciferrii*, HP2; C) *P. cookei*, HP32; D) *P. blaschkeae*, HP4.

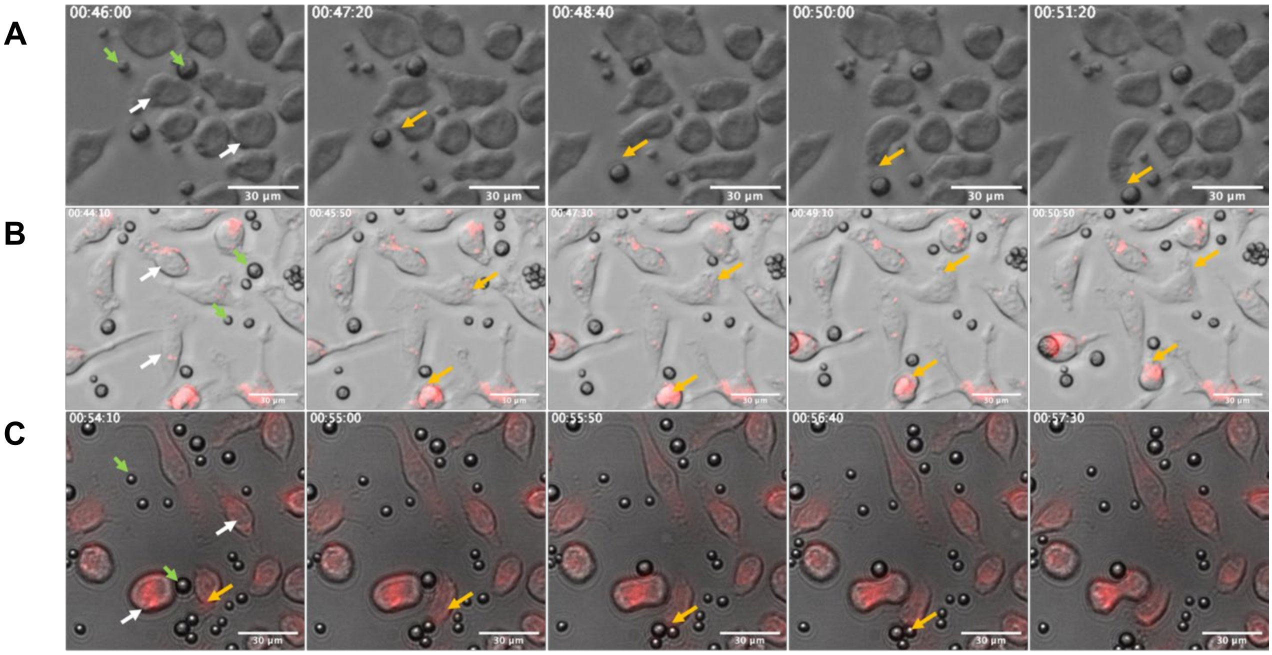

Sup. Fig. 4 Chemotaxis of J774A.1 cells towards human-associated species of *Prototheca*. Algal cells are indicated by green arrows; macrophages are indicated by white arrows; suspected chemotaxis is indicated by yellow arrows. LTR staining indicates acidified compartments within the macrophages – indicating a lack of acidified phagolysosomes containing phagocytosed algal cells. Rows show selected frames from a single video, at times indicated by the time stamp in the top left. The species and strain of the algal cells in each row are A) *P. cutis*, HP28; B) *P. paracutis*, HP31; C) *P. wickerhamii*, HP52.

A

B

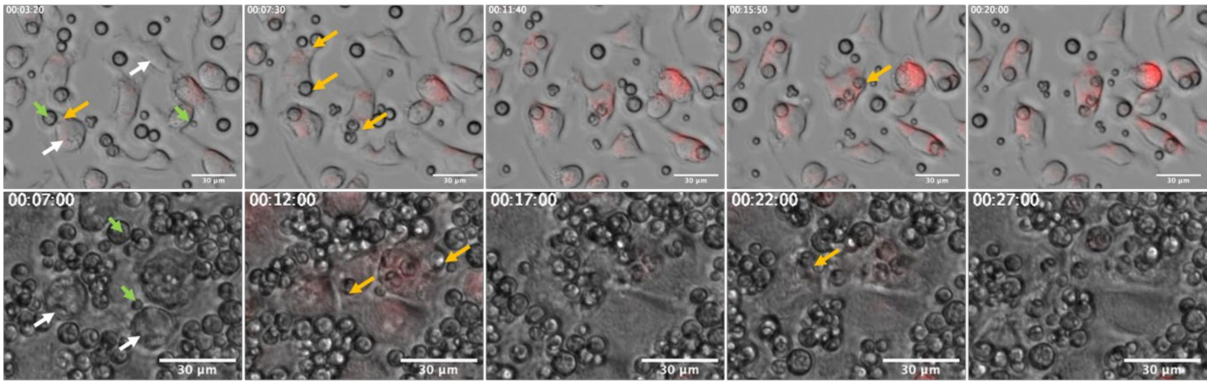

Sup. Fig. 5 Phagocytosis of *P. miyajii*, HP27, by macrophages. Algal cells are indicated by green arrows; macrophages are indicated by white arrows; phagocytosis events are indicated by yellow arrows. LTR staining indicates acidified compartments within the macrophages – which could not reliably identify acidified phagolysosomes containing phagocytosed algal cells. Rows show selected frames from videos, at times indicated by the time stamp in the top left. Algal cells were exposed to: A) J774A.1 cells; B) HMDMs from donor 2.

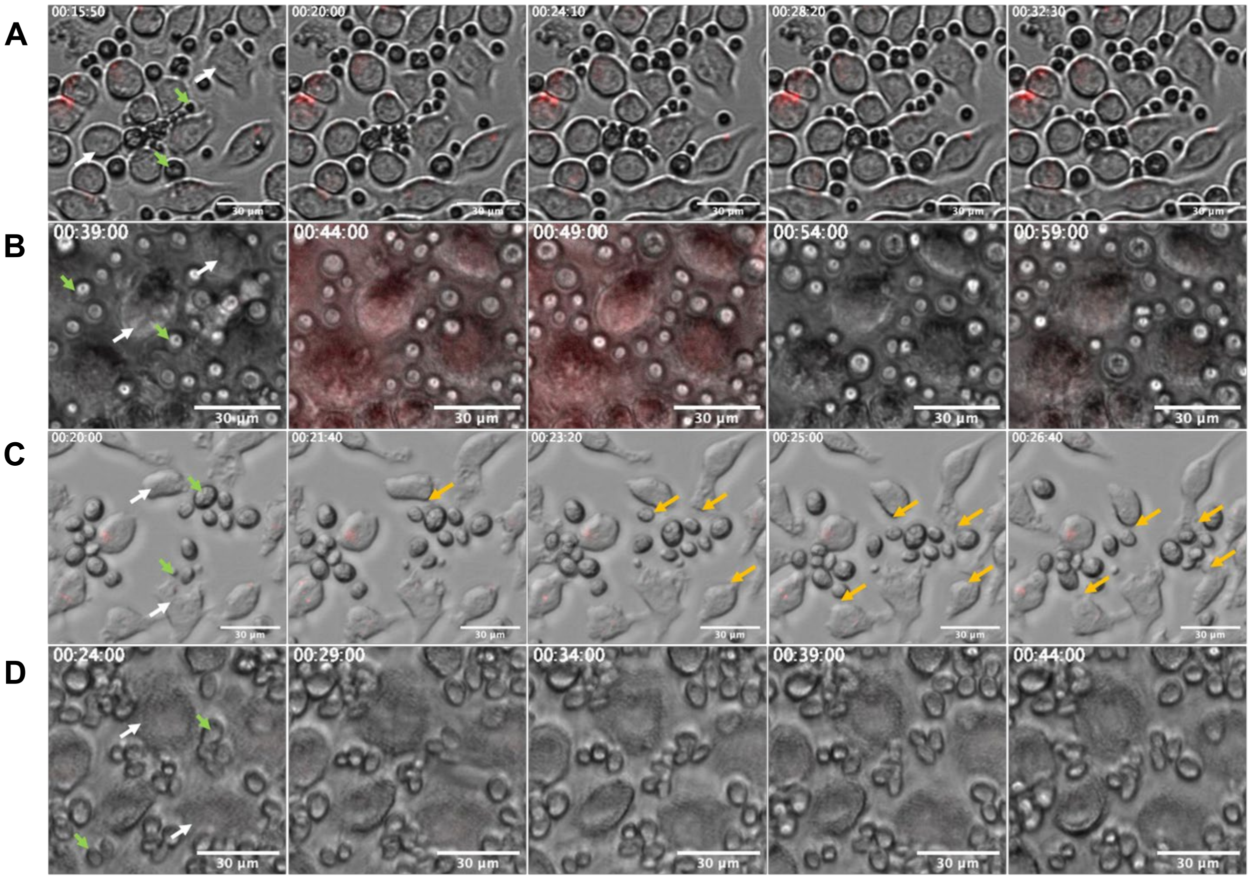

Sup. Fig. 6 A lack of phagocytosis of true environmental species of *Prototheca* by macrophages. Algal cells are indicated by green arrows; macrophages are indicated by white arrows; suspected chemotaxis is indicated by yellow arrows. LTR staining indicates acidified compartments within the macrophages – indicating a lack of acidified phagolysosomes containing phagocytosed algal cells. Rows show selected frames from a single video, at times indicated by the time stamp in the top left. The species and strain of the algal cells in each row are A) *P. tumulicola*, HP29, in J774A.1 cells; B) *P. tumulicola*, HP29, in HMDMs from donor 2; C) *P. moriformis*, HP51, in J774A.1 cells; D) *P. moriformis*, HP51, in HMDMs from donor 1.

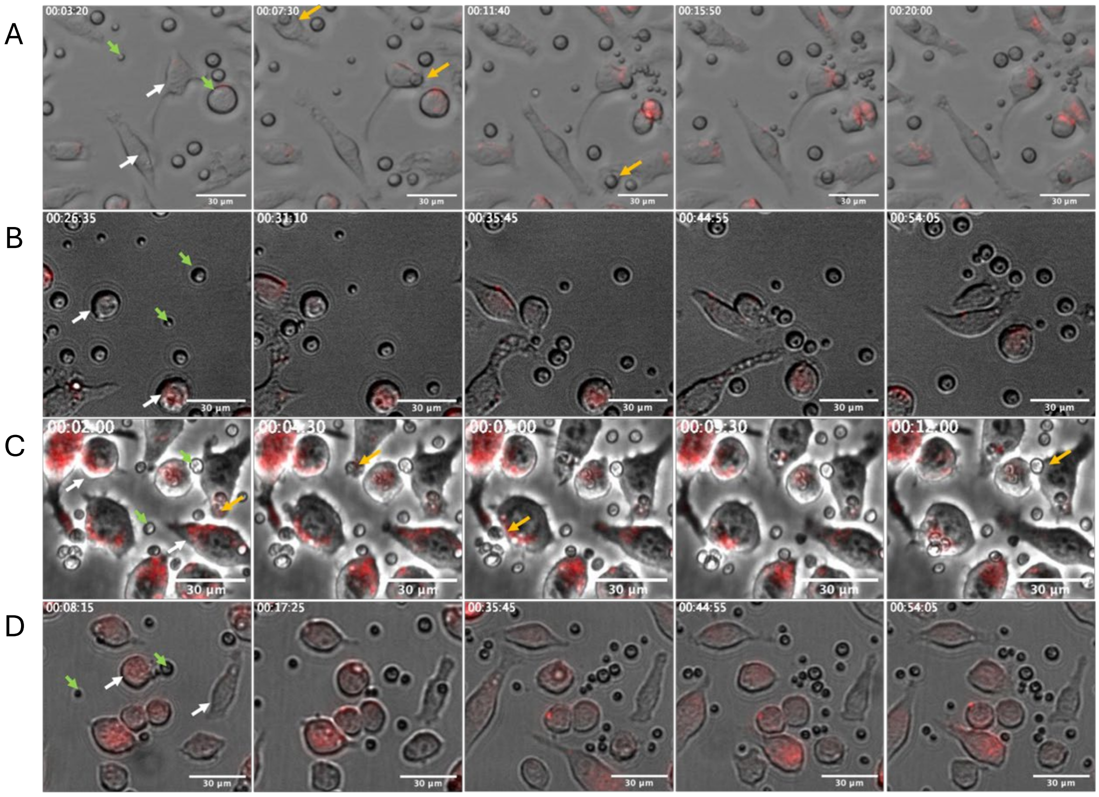

Sup. Fig. 7 Inconsistent phagocytosis of strains of *Auxenochlorella* by J774A.1 cells. Algal cells are indicated by green arrows; macrophages are indicated by white arrows; phagocytosis events are indicated by yellow arrows. LTR staining indicates acidified compartments within the macrophages – which could not reliably identify acidified phagolysosomes containing phagocytosed algal cells. Rows show selected frames from videos, at times indicated by the time stamp in the top left. The species and strain of the algal cells in each row are (A and B) *A. protothecoides*, HA1; (C and D) *Auxenochlorella* sp. 1, HA6. Phagocytosis is observed in rows A and C and is not observed in rows B and D.

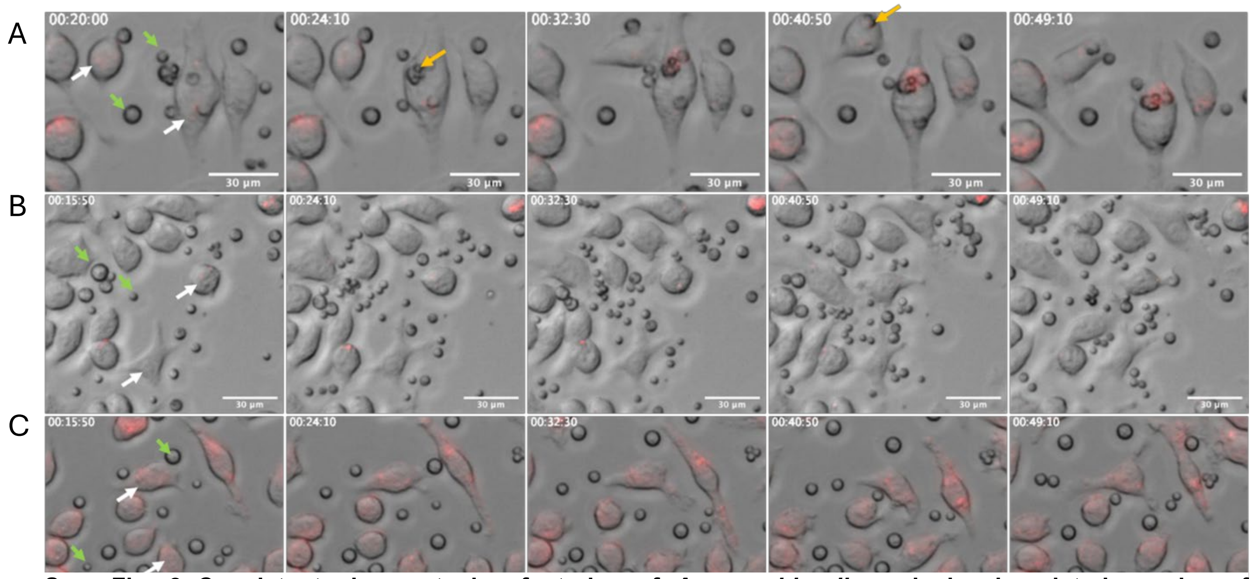

Sup. Fig. 8 Consistent phagocytosis of strains of *Auxenochlorella* and closely related species of *Prototheca* by J774A.1 cells. Algal cells are indicated by green arrows; macrophages are indicated by white arrows; phagocytosis events are indicated by yellow arrows. LTR staining indicates acidified compartments within the macrophages – which could not reliably identify acidified phagolysosomes containing phagocytosed algal cells. Rows show selected frames from videos, at times indicated by the time stamp in the top left. The species and strain of the algal cells in each row are A) *P. xanthoriae*, HP54; B) *A. symbiontica*, HA5; C) *Auxenochlorella* sp. 2, HA7.

Sup. Table 1 Pairwise comparisons of mean phagocytic index within AHP sub-lineages.

Tukey's multiple comparisons test	**J774A.1**					**HMDMs**				
**Group 1**	Mean Diff.	95.00% CI of diff.	Below threshold?	Summary	Adjusted P Value	Mean Diff.	95.00% CI of diff.	Below threshold?	Summary	Adjusted P Value
P. bovis vs. P. ciferii	-16.69	-50.17 to 16.79	No	ns	0.8717	-4.933	-64.06 to 54.19	No	ns	>0.9999
P. bovis vs. P. blaschkeae	-7.333	-44.76 to 30.10	No	ns	>0.9999	-37.6	-105.3 to 30.13	No	ns	0.7407
P. bovis vs. P. cookei	-9.333	-42.81 to 24.15	No	ns	0.9991	-14.6	-82.33 to 53.13	No	ns	>0.9999
P. ciferii vs. P. blaschkeae	9.36	-28.07 to 46.79	No	ns	0.9997	-32.67	-106.6 to 41.24	No	ns	0.9304
P. ciferii vs. P. cookei	7.36	-26.12 to 40.84	No	ns	>0.9999	-9.667	-83.57 to 64.24	No	ns	>0.9999
P. blaschkeae vs. P. cookei	-2	-39.43 to 35.43	No	ns	>0.9999	23	-57.96 to 104.0	No	ns	0.9985
**Group 2**	Mean Diff.	95.00% CI of diff.	Below threshold?	Summary	Adjusted P Value	Mean Diff.	95.00% CI of diff.	Below threshold?	Summary	Adjusted P Value
P. cutis vs. P. miyajii	-58	-91.48 to -24.52	**Yes**	****	<0.0001	-10.67	-76.77 to 55.43	No	ns	>0.9999
P. cutis vs. P. wickerhamii	2.443	-31.04 to 35.92	No	ns	>0.9999	-4	-63.12 to 55.12	No	ns	>0.9999
P. cutis vs. P. paracutis	-19.33	-52.81 to 14.15	No	ns	0.7183	5.335	-68.57 to 79.24	No	ns	>0.9999
P. miyajii vs. P. wickerhamii	60.44	26.96 to 93.92	Yes	****	<0.0001	6.667	-52.46 to 65.79	No	ns	>0.9999
P. miyajii vs. P. paracutis	38.67	5.188 to 72.15	Yes	*	0.0121	16	-57.90 to 89.90	No	ns	>0.9999
P. wickerhamii vs. P. paracutis	-21.78	-55.26 to 11.70	No	ns	0.5451	9.335	-58.40 to 77.07	No	ns	>0.9999
**Group 3**	Mean Diff.	95.00% CI of diff.	Below threshold?	Summary	Adjusted P Value	Mean Diff.	95.00% CI of diff.	Below threshold?	Summary	Adjusted P Value
P. tumulicola vs. P. moriformis	-2.63	-36.11 to 30.85	No	ns	>0.9999	1	-79.96 to 81.96	No	ns	>0.9999
**Group 4**	Mean Diff.	95.00% CI of diff.	Below threshold?	Summary	Adjusted P Value	Mean Diff.	95.00% CI of diff.	Below threshold?	Summary	Adjusted P Value
P. xanthoriae vs. A. protothecoides	24.33	-2.137 to 50.80	No	ns	0.0981	25.17	-36.67 to 87.00	No	ns	0.9617
P. xanthoriae vs. A. symbiontica	45.21	16.22 to 74.21	**Yes**	***	0.0002	24.5	-45.61 to 94.61	No	ns	0.9891
P. xanthoriae vs. A. sp. 1	35.83	6.836 to 64.82	**Yes**	**	0.0055	6.5	-63.61 to 76.61	No	ns	>0.9999
P. xanthoriae vs. A. sp. 2	46.5	17.50 to 75.49	**Yes**	***	0.0001	38.5	-52.01 to 129.0	No	ns	0.9469
A. protothecoides vs. A. symbiontica	20.88	-10.43 to 52.20	No	ns	0.5059	-0.6667	-74.57 to 73.24	No	ns	>0.9999
A. protothecoides vs. A. sp. 1	11.5	-19.82 to 42.82	No	ns	0.9871	-18.67	-92.57 to 55.24	No	ns	0.9996
A. protothecoides vs. A. sp. 2	22.17	-9.150 to 53.48	No	ns	0.4117	13.33	-80.15 to 106.8	No	ns	>0.9999
A. symbiontica vs. A. sp. 1	-9.383	-42.86 to 24.10	No	ns	0.9991	-18	-98.96 to 62.96	No	ns	0.9999
A. symbiontica vs. A. sp. 2	1.283	-32.20 to 34.76	No	ns	>0.9999	14	-85.15 to 113.2	No	ns	>0.9999
A. sp. 1 *vs. A.* sp. 2	10.67	-22.81 to 44.15	No	ns	0.9966	32	-67.15 to 131.2	No	ns	0.9947

Sup. Table 2 Pairwise comparisons of mean uptake within AHP sub-lineages.

Tukey's multiple comparisons test	**J774A.1**					**HMDMs**				
**Group 1**	Mean Diff.	95.00% CI of diff.	Below threshold?	Summary	Adjusted P Value	Mean Diff.	95.00% CI of diff.	Below threshold?	Summary	Adjusted P Value
P. bovis vs. P. ciferii	-2.435	-3.007 to -1.863	**Yes**	****	<0.0001	0.232	-0.5596 to 1.024	No	ns	0.9997
P. bovis vs. P. blaschkeae	1.26	0.6182 to 1.902	**Yes**	****	<0.0001	-1.049	-1.960 to -0.1392	**Yes**	**	0.0081
P. bovis vs. P. cookei	-0.4733	-1.047 to 0.1007	No	ns	0.2477	0.212	-0.6949 to 1.119	No	ns	>0.9999
P. ciferii vs. P. blaschkeae	3.695	3.055 to 4.335	**Yes**	****	<0.0001	-1.281	-2.274 to -0.2889	**Yes**	**	0.0012
P. ciferii vs. P. cookei	1.962	1.390 to 2.534	**Yes**	****	<0.0001	-0.02	-1.010 to 0.9696	No	ns	>0.9999
P. blaschkeae vs. P. cookei	-1.733	-2.375 to -1.092	**Yes**	****	<0.0001	1.261	0.1747 to 2.348	**Yes**	**	0.0073
**Group 2**	Mean Diff.	95.00% CI of diff.	Below threshold?	Summary	Adjusted P Value	Mean Diff.	95.00% CI of diff.	Below threshold?	Summary	Adjusted P Value
P. cutis vs. P. miyajii	-1.027	-1.601 to -0.4526	**Yes**	****	<0.0001	-0.5533	-1.438 to 0.3318	No	ns	0.7196
P. cutis vs. P. wickerhamii	0.02762	-0.4575 to 0.5128	No	ns	>0.9999	-0.05333	-0.8450 to 0.7383	No	ns	>0.9999
P. cutis vs. P. paracutis	-0.2133	-0.7874 to 0.3607	No	ns	0.9955	0.1167	-0.8729 to 1.106	No	ns	>0.9999
P. miyajii vs. P. wickerhamii	1.054	0.5691 to 1.539	**Yes**	****	<0.0001	0.5	-0.2916 to 1.292	No	ns	0.7045
P. miyajii vs. P. paracutis	0.8133	0.2393 to 1.387	**Yes**	***	0.0002	0.67	-0.3196 to 1.660	No	ns	0.5914
P. wickerhamii vs. P. paracutis	-0.241	-0.7261 to 0.2442	No	ns	0.9375	0.17	-0.7369 to 1.077	No	ns	>0.9999
**Group 3**	Mean Diff.	95.00% CI of diff.	Below threshold?	Summary	Adjusted P Value	Mean Diff.	95.00% CI of diff.	Below threshold?	Summary	Adjusted P Value
P. tumulicola vs. P. moriformis	-0.03268	-0.6039 to 0.5385	No	ns	>0.9999	0	-1.084 to 1.084	No	ns	>0.9999
**Group 4**	Mean Diff.	95.00% CI of diff.	Below threshold?	Summary	Adjusted P Value	Mean Diff.	95.00% CI of diff.	Below threshold?	Summary	Adjusted P Value
P. xanthoriae vs. A. protothecoides	0.4672	0.01374 to 0.9207	**Yes**	*	0.0359	1.527	0.6987 to 2.355	**Yes**	****	<0.0001
P. xanthoriae vs. A. symbiontica	0.8009	0.3063 to 1.296	**Yes**	****	<0.0001	1.33	0.3912 to 2.269	**Yes**	***	0.0002
P. xanthoriae vs. A. sp. 1	0.5806	0.08373 to 1.077	**Yes**	**	0.0066	0.8	-0.1388 to 1.739	No	ns	0.2
P. xanthoriae vs. A. sp. 2	0.8139	0.3171 to 1.311	**Yes**	****	<0.0001	1.96	0.7480 to 3.172	**Yes**	****	<0.0001
A. protothecoides vs. A. symbiontica	0.3337	-0.2012 to 0.8686	No	ns	0.7233	-0.1967	-1.186 to 0.7929	No	ns	>0.9999
A. protothecoides vs. A. sp. 1	0.1133	-0.4236 to 0.6503	No	ns	>0.9999	-0.7267	-1.716 to 0.2629	No	ns	0.4456
A. protothecoides vs. A. sp. 2	0.3467	-0.1903 to 0.8836	No	ns	0.671	0.4333	-0.8184 to 1.685	No	ns	0.9978
A. symbiontica vs. A. sp. 1	-0.2204	-0.7925 to 0.3518	No	ns	0.9935	-0.53	-1.614 to 0.5540	No	ns	0.9446
A. symbiontica vs. A. sp. 2	0.01298	-0.5591 to 0.5851	No	ns	>0.9999	0.63	-0.6976 to 1.958	No	ns	0.9564
A. sp. *1 vs. A.* sp. 2	0.2333	-0.3407 to 0.8074	No	ns	0.989	1.16	-0.1676 to 2.488	No	ns	0.1676

Sup. Table 3 Pairwise comparisons of mean displacement within AHP sub-linages.

Tukey's multiple comparisons test	**J774A.1**					**HMDMs**				
**Group 1**	Mean Diff.	95.00% CI of diff.	Below threshold?	Summary	Adjusted P Value	Mean Diff.	95.00% CI of diff.	Below threshold?	Summary	Adjusted P Value
P. bovis vs. P. ciferii	1.123	-0.3927 to 2.639	No	ns	0.4309	2.944	1.776 to 4.111	**Yes**	****	<0.0001
P. bovis vs. P. blaschkeae	-1.384	-2.912 to 0.1452	No	ns	0.1279	5.47	4.175 to 6.766	**Yes**	****	<0.0001
P. bovis vs. P. cookei	1.175	-0.2382 to 2.588	No	ns	0.2359	6.997	5.577 to 8.416	**Yes**	****	<0.0001
P. ciferii vs. P. blaschkeae	-2.507	-4.013 to -0.9998	**Yes**	****	<0.0001	2.527	1.041 to 4.012	**Yes**	****	<0.0001
P. ciferii vs. P. cookei	0.052	-1.337 to 1.441	No	ns	>0.9999	4.053	2.459 to 5.647	**Yes**	****	<0.0001
P. blaschkeae vs. P. cookei	2.559	1.155 to 3.962	**Yes**	****	<0.0001	1.526	-0.1640 to 3.217	No	ns	0.1303
**Group 2**	Mean Diff.	95.00% CI of diff.	Below threshold?	Summary	Adjusted P Value	Mean Diff.	95.00% CI of diff.	Below threshold?	Summary	Adjusted P Value
P. cutis vs. P. miyajii	-2.118	-3.333 to -0.9034	**Yes**	****	<0.0001	-4.559	-6.368 to -2.750	**Yes**	****	<0.0001
P. cutis vs. P. wickerhamii	3.255	2.153 to 4.356	**Yes**	****	<0.0001	-2.643	-4.129 to -1.157	**Yes**	****	<0.0001
P. cutis vs. P. paracutis	-0.2304	-1.497 to 1.037	No	ns	>0.9999	-0.9972	-2.874 to 0.8794	No	ns	0.8977
P. miyajii vs. P. wickerhamii	5.373	4.346 to 6.401	**Yes**	****	<0.0001	1.916	0.2885 to 3.543	**Yes**	**	0.0058
P. miyajii vs. P. paracutis	1.888	0.6851 to 3.091	**Yes**	****	<0.0001	3.561	1.571 to 5.552	**Yes**	****	<0.0001
P. wickerhamii vs. P. paracutis	-3.485	-4.574 to -2.397	**Yes**	****	<0.0001	1.646	-0.05659 to 3.348	No	ns	0.0707
**Group 3**	Mean Diff.	95.00% CI of diff.	Below threshold?	Summary	Adjusted P Value	Mean Diff.	95.00% CI of diff.	Below threshold?	Summary	Adjusted P Value
P. tumulicola vs. P. moriformis	2.871	0.7406 to 5.002	**Yes**	***	0.0005	-10.29	-12.44 to -8.144	**Yes**	****	<0.0001
**Group 4**	Mean Diff.	95.00% CI of diff.	Below threshold?	Summary	Adjusted P Value	Mean Diff.	95.00% CI of diff.	Below threshold?	Summary	Adjusted P Value
P. xanthoriae vs. A. protothecoides	0.2725	-0.7709 to 1.316	No	ns	>0.9999	-1.827	-3.260 to -0.3945	**Yes**	**	0.0015
P. xanthoriae vs. A. symbiontica	0.05615	-1.208 to 1.320	No	ns	>0.9999	-1.45	-3.218 to 0.3184	No	ns	0.2571
P. xanthoriae vs. A. sp. 1	1.978	0.5079 to 3.448	**Yes**	***	0.0005	-1.616	-3.193 to -0.03835	**Yes**	*	0.0383
P. xanthoriae vs. A. sp. 2	1.284	0.1966 to 2.371	**Yes**	**	0.0055	-1.957	-4.038 to 0.1238	No	ns	0.0918
A. protothecoides vs. A. symbiontica	-0.2163	-1.573 to 1.140	No	ns	>0.9999	0.3774	-1.394 to 2.148	No	ns	>0.9999
A. protothecoides vs. A. sp. 1	1.705	0.1556 to 3.255	**Yes**	*	0.0157	0.2113	-1.369 to 1.792	No	ns	>0.9999
A. protothecoides vs. A. sp. 2	1.011	-0.1816 to 2.204	No	ns	0.2079	-0.1298	-2.213 to 1.953	No	ns	>0.9999
A. symbiontica vs. A. sp. 1	1.922	0.2153 to 3.628	**Yes**	*	0.0114	-0.1661	-2.056 to 1.724	No	ns	>0.9999
A. symbiontica vs. A. sp. 2	1.228	-0.1627 to 2.618	No	ns	0.1553	-0.5071	-2.834 to 1.819	No	ns	>0.9999
A. sp. 1 *vs. A.* sp. 2	-0.6942	-2.274 to 0.8855	No	ns	0.9778	-0.341	-2.526 to 1.844	No	ns	>0.9999

Sup. Table 4 Pairwise comparisons of mean speed within AHP sub-lineages.

Tukey's multiple comparisons test	**J774A.1**					**HMDMs**				
**Group 1**	Mean Diff.	95.00% CI of diff.	Below threshold?	Summary	Adjusted P Value	Mean Diff.	95.00% CI of diff.	Below threshold?	Summary	Adjusted P Value
P. bovis vs. P. ciferii	0.008514	0.003857 to 0.01317	**Yes**	****	<0.0001	4.351	3.817 to 4.884	**Yes**	****	<0.0001
P. bovis vs. P. blaschkeae	0.004827	0.0001237 to 0.009531	**Yes**	*	0.0375	3.919	3.499 to 4.340	**Yes**	****	<0.0001
P. bovis vs. P. cookei	0.003396	-0.0008905 to 0.007683	No	ns	0.3127	3.43	2.970 to 3.891	**Yes**	****	<0.0001
P. ciferii vs. P. blaschkeae	-0.00369	-0.008689 to 0.001315	No	ns	0.4404	-0.4312	-1.042 to 0.1798	No	ns	0.5193
P. ciferii vs. P. cookei	-0.00512	-0.009730 to -0.0005054	**Yes**	*	0.014	-0.9203	-1.560 to -0.2810	**Yes**	***	0.0001
P. blaschkeae vs. P. cookei	-0.00143	-0.006091 to 0.003229	No	ns	0.9994	-0.4891	-1.038 to 0.05933	No	ns	0.1438
**Group 2**	Mean Diff.	95.00% CI of diff.	Below threshold?	Summary	Adjusted P Value	Mean Diff.	95.00% CI of diff.	Below threshold?	Summary	Adjusted P Value
P. cutis vs. P. miyajii	-0.01399	-0.01802 to -0.009954	**Yes**	****	<0.0001	-1.227	-1.814 to -0.6405	**Yes**	****	<0.0001
P. cutis vs. P. wickerhamii	0.002836	-0.0008201 to 0.006493	No	ns	0.3489	-1.555	-2.038 to -1.073	**Yes**	****	<0.0001
P. cutis vs. P. paracutis	-0.02829	-0.03250 to -0.02409	**Yes**	****	<0.0001	-0.1989	-0.8078 to 0.4099	No	ns	0.9989
P. miyajii vs. P. wickerhamii	0.01682	0.01341 to 0.02023	**Yes**	****	<0.0001	-0.3279	-0.8559 to 0.2000	No	ns	0.7307
P. miyajii vs. P. paracutis	-0.01431	-0.01830 to -0.01031	**Yes**	****	<0.0001	1.028	0.3827 to 1.674	**Yes**	****	<0.0001
P. wickerhamii vs. P. paracutis	-0.03113	-0.03474 to -0.02752	**Yes**	****	<0.0001	1.356	0.8041 to 1.909	**Yes**	****	<0.0001
**Group 3**	Mean Diff.	95.00% CI of diff.	Below threshold?	Summary	Adjusted P Value	Mean Diff.	95.00% CI of diff.	Below threshold?	Summary	Adjusted P Value
P. tumulicola vs. P. moriformis	-0.00511	-0.01157 to 0.001345	No	ns	0.3141	2.024	1.434 to 2.614	**Yes**	****	<0.0001
**Group 4**	Mean Diff.	95.00% CI of diff.	Below threshold?	Summary	Adjusted P Value	Mean Diff.	95.00% CI of diff.	Below threshold?	Summary	Adjusted P Value
P. xanthoriae vs. A. protothecoides	0.008861	0.005397 to 0.01232	**Yes**	****	<0.0001	-0.8747	-1.340 to -0.4099	**Yes**	****	<0.0001
P. xanthoriae vs. A. symbiontica	0.00744	0.003243 to 0.01164	**Yes**	****	<0.0001	-1.337	-1.911 to -0.7636	**Yes**	****	<0.0001
P. xanthoriae vs. A. sp. *1*	0.01464	0.009763 to 0.01952	**Yes**	****	<0.0001	-0.6397	-1.151 to -0.1279	**Yes**	**	0.0021
P. xanthoriae vs. A. sp. *2*	0.01246	0.008855 to 0.01607	**Yes**	****	<0.0001	-1.938	-2.613 to -1.263	**Yes**	****	<0.0001
A. protothecoides vs. A. symbiontica	-0.00142	-0.005923 to 0.003081	No	ns	0.9992	-0.4625	-1.037 to 0.1121	No	ns	0.2864
A. protothecoides vs. A. sp. *1*	0.005782	0.0006371 to 0.01093	**Yes**	*	0.0117	0.2351	-0.2778 to 0.7479	No	ns	0.9678
A. protothecoides vs. A. sp. *2*	0.003603	-0.0003572 to 0.007563	No	ns	0.1225	-1.063	-1.739 to -0.3872	**Yes**	****	<0.0001
A. symbiontica vs. A. sp. *1*	0.007203	0.001539 to 0.01287	**Yes**	**	0.0015	0.6975	0.08432 to 1.311	**Yes**	**	0.0098
A. symbiontica vs. A. sp. *2*	0.005024	0.0004088 to 0.009639	**Yes**	*	0.0181	-0.6006	-1.355 to 0.1543	No	ns	0.3056
A. sp. *1 vs. A.* sp. *2*	-0.00218	-0.007423 to 0.003065	No	ns	0.9867	-1.298	-2.007 to -0.5891	**Yes**	****	<0.0001

Sup. Table 5 Pairwise comparisons of linearity of forward progression within AHP sub-lineages.

Tukey's multiple comparisons test	**J774A.1**					**HMDMs**				
**Group 1**	Mean Diff.	95.00% CI of diff.	Below threshold?	Summary	Adjusted P Value	Mean Diff.	95.00% CI of diff.	Below threshold?	Summary	Adjusted P Value
P. bovis vs. P. ciferii	0.01458	-0.02808 to 0.05724	No	ns	>0.9999	0.0472	0.005618 to 0.08879	**Yes**	*	0.0101
P. bovis vs. P. blaschkeae	-0.02089	-0.06395 to 0.02217	No	ns	>0.9999	0.04023	-0.005836 to 0.08629	No	ns	0.1687
P. bovis vs. P. cookei	-0.09068	-0.1299 to -0.05141	**Yes**	****	<0.0001	0.07408	0.02340 to 0.1248	**Yes**	****	<0.0001
P. ciferii vs. P. blaschkeae	-0.03546	-0.08125 to 0.01033	No	ns	0.5179	-0.006977	-0.05979 to 0.04584	No	ns	>0.9999
P. ciferii vs. P. cookei	-0.1053	-0.1475 to -0.06300	**Yes**	****	<0.0001	0.02688	-0.03001 to 0.08377	No	ns	0.9585
P. blaschkeae vs. P. cookei	-0.06979	-0.1124 to -0.02714	**Yes**	****	<0.0001	0.03386	-0.02639 to 0.09410	No	ns	0.8505
**Group 2**	Mean Diff.	95.00% CI of diff.	Below threshold?	Summary	Adjusted P Value	Mean Diff.	95.00% CI of diff.	Below threshold?	Summary	Adjusted P Value
P. cutis vs. P. miyajii	-0.004663	-0.04162 to 0.03230	No	ns	>0.9999	-0.1198	-0.1736 to -0.06593	**Yes**	****	<0.0001
P. cutis vs. P. wickerhamii	0.0199	-0.01357 to 0.05338	No	ns	0.9831	-0.06809	-0.1211 to -0.01508	**Yes**	**	0.0013
P. cutis vs. P. paracutis	-0.01043	-0.04893 to 0.02807	No	ns	>0.9999	-0.02046	-0.08738 to 0.04647	No	ns	0.9995
P. miyajii vs. P. wickerhamii	0.02457	-0.006677 to 0.05581	No	ns	0.4744	0.05167	0.005813 to 0.09754	**Yes**	*	0.0113
P. miyajii vs. P. paracutis	-0.005771	-0.04235 to 0.03080	No	ns	>0.9999	0.09931	0.03789 to 0.1607	**Yes**	****	<0.0001
P. wickerhamii vs. P. paracutis	-0.03034	-0.06339 to 0.002712	No	ns	0.134	0.04764	-0.01306 to 0.1083	No	ns	0.3286
**Group 3**	Mean Diff.	95.00% CI of diff.	Below threshold?	Summary	Adjusted P Value	Mean Diff.	95.00% CI of diff.	Below threshold?	Summary	Adjusted P Value
P. tumulicola vs. P. moriformis	-0.01051	-0.06961 to 0.04859	No	ns	>0.9999	0.007869	-0.05686 to 0.07259	No	ns	>0.9999
**Group 4**	Mean Diff.	95.00% CI of diff.	Below threshold?	Summary	Adjusted P Value	Mean Diff.	95.00% CI of diff.	Below threshold?	Summary	Adjusted P Value
P. xanthoriae vs. A. protothecoides	-0.01458	-0.04631 to 0.01715	No	ns	>0.9999	-0.03028	-0.08134 to 0.02077	No	ns	0.7917
P. xanthoriae vs. A. symbiontica	-0.03911	-0.07753 to -0.0006818	**Yes**	*	0.0398	-0.0381	-0.1011 to 0.02490	No	ns	0.7673
P. xanthoriae vs. A. sp. 1	0.02222	-0.02253 to 0.06697	No	ns	0.9999	-0.01863	-0.07493 to 0.03768	No	ns	0.9987
P. xanthoriae vs. A. sp. 2	0.02288	-0.01020 to 0.05595	No	ns	0.8134	0.06908	-0.005005 to 0.1432	No	ns	0.0991
A. protothecoides vs. A. symbiontica	-0.02453	-0.06575 to 0.01669	No	ns	0.9828	-0.007812	-0.07091 to 0.05528	No	ns	>0.9999
A. protothecoides vs. A. sp. 1	0.0368	-0.01037 to 0.08397	No	ns	0.4969	0.01166	-0.04476 to 0.06807	No	ns	>0.9999
A. protothecoides vs. A. sp. 2	0.03745	0.001168 to 0.07374	**Yes**	*	0.033	0.09936	0.02519 to 0.1735	**Yes**	***	0.0005
A. symbiontica vs. A. sp. 1	0.06133	0.009417 to 0.1132	**Yes**	**	0.004	0.01947	-0.04794 to 0.08688	No	ns	0.9997
A. symbiontica vs. A. sp. 2	0.06198	0.01972 to 0.1043	**Yes**	****	<0.0001	0.1072	0.02433 to 0.1900	**Yes**	**	0.0011
A. sp. 1 vs. *A.* sp. 2	0.000654	-0.04743 to 0.04874	No	ns	>0.9999	0.0877	0.009831 to 0.1656	**Yes**	*	0.0114

Sup. Table 6 Pairwise comparisons of mean directional change rate within AHP sub-lineages.

Tukey's multiple comparisons test	**J774A.1**					**HMDMs**				
**Group 1**	Mean Diff.	95.00% CI of diff.	Below threshold?	Summary	Adjusted P Value	Mean Diff.	95.00% CI of diff.	Below threshold?	Summary	Adjusted P Value
P. bovis vs. P. ciferii	0.000418	-0.001192 to 0.002028	No	ns	>0.9999	0.508	0.4340 to 0.5820	**Yes**	****	<0.0001
P. bovis vs. P. blaschkeae	-9.36E-05	-0.001725 to 0.001538	No	ns	>0.9999	0.1972	0.1054 to 0.2889	**Yes**	****	<0.0001
P. bovis vs. P. cookei	0.004911	0.003340 to 0.006481	**Yes**	****	<0.0001	-0.00903	-0.1114 to 0.09331	No	ns	>0.9999
P. ciferii vs. P. blaschkeae	-0.0005116	-0.002238 to 0.001215	No	ns	0.9996	-0.3108	-0.4077 to -0.2139	**Yes**	****	<0.0001
P. ciferii vs. P. cookei	0.004493	0.002823 to 0.006162	**Yes**	****	<0.0001	-0.517	-0.6240 to -0.4101	**Yes**	****	<0.0001
P. blaschkeae vs. P. cookei	0.005004	0.003314 to 0.006694	**Yes**	****	<0.0001	-0.2062	-0.3261 to -0.08625	**Yes**	****	<0.0001
**Group 2**	Mean Diff.	95.00% CI of diff.	Below threshold?	Summary	Adjusted P Value	Mean Diff.	95.00% CI of diff.	Below threshold?	Summary	Adjusted P Value
P. cutis vs. P. miyajii	-0.003147	-0.004578 to -0.001716	**Yes**	****	<0.0001	0.1182	0.01448 to 0.2220	**Yes**	**	0.0096
P. cutis vs. P. wickerhamii	-0.01056	-0.01188 to -0.009246	**Yes**	****	<0.0001	-0.01041	-0.1149 to 0.09411	No	ns	>0.9999
P. cutis vs. P. paracutis	-0.008141	-0.009646 to -0.006635	**Yes**	****	<0.0001	9.34E-05	-0.1311 to 0.1313	No	ns	>0.9999
P. miyajii vs. P. wickerhamii	-0.007416	-0.008623 to -0.006209	**Yes**	****	<0.0001	-0.1286	-0.2177 to -0.03963	**Yes**	****	<0.0001
P. miyajii vs. P. paracutis	-0.004993	-0.006403 to -0.003584	**Yes**	****	<0.0001	-0.1181	-0.2374 to 0.001110	No	ns	0.0552
P. wickerhamii vs. P. paracutis	0.002423	0.001128 to 0.003717	**Yes**	****	<0.0001	0.0105	-0.1094 to 0.1304	No	ns	>0.9999
**Group 3**	Mean Diff.	95.00% CI of diff.	Below threshold?	Summary	Adjusted P Value	Mean Diff.	95.00% CI of diff.	Below threshold?	Summary	Adjusted P Value
P. tumulicola vs. P. moriformis	0.002842	0.0006237 to 0.005061	**Yes**	**	0.0013	0.1445	0.01599 to 0.2730	**Yes**	*	0.0116
**Group 4**	Mean Diff.	95.00% CI of diff.	Below threshold?	Summary	Adjusted P Value	Mean Diff.	95.00% CI of diff.	Below threshold?	Summary	Adjusted P Value
P. xanthoriae vs. A. protothecoides	0.002186	0.0009762 to 0.003396	**Yes**	****	<0.0001	0.03312	-0.06951 to 0.1358	No	ns	0.999
P. xanthoriae vs. A. symbiontica	0.003995	0.002519 to 0.005472	**Yes**	****	<0.0001	0.04552	-0.08159 to 0.1726	No	ns	0.997
P. xanthoriae vs. A. sp. *1*	0.0005734	-0.001136 to 0.002283	No	ns	0.9985	0.03697	-0.07503 to 0.1490	No	ns	0.9987
P. xanthoriae vs. A. sp. *2*	0.001878	0.0006221 to 0.003134	**Yes**	****	<0.0001	-0.248	-0.3964 to -0.09949	**Yes**	****	<0.0001
A. protothecoides vs. A. symbiontica	0.001809	0.0002308 to 0.003388	**Yes**	**	0.0088	0.0124	-0.1156 to 0.1404	No	ns	>0.9999
A. protothecoides vs. A. sp. *1*	-0.001613	-0.003411 to 0.0001859	No	ns	0.1379	0.003845	-0.1092 to 0.1169	No	ns	>0.9999
A. protothecoides vs. A. sp. *2*	-0.0003079	-0.001683 to 0.001067	No	ns	>0.9999	-0.2811	-0.4303 to -0.1318	**Yes**	****	<0.0001
A. symbiontica vs. A. sp. *1*	-0.003422	-0.005409 to -0.001434	**Yes**	****	<0.0001	-0.008556	-0.1442 to 0.1271	No	ns	>0.9999
A. symbiontica vs. A. sp. *2*	-0.002117	-0.003732 to -0.0005030	**Yes**	***	0.0008	-0.2935	-0.4605 to -0.1265	**Yes**	****	<0.0001
A. sp. *1 vs. A.* sp. *2*	0.001305	-0.0005252 to 0.003134	No	ns	0.501	-0.2849	-0.4408 to -0.1291	**Yes**	****	<0.0001
