# Supplementary material for: Phylogenetic analysis of pathogenic algae reveals lineage-dependent patterns of phagocytosis
Source: mBio. 2025 Apr 30;16(6):e00498-25. doi: 10.1128/mbio.00498-25 (PMC12153285; doi:10.1128/mbio.00498-25)
Supplement: Movie legends — Legends for the two supplemental movies. [file mbio.00498-25-s0002.docx]

**Supplementary Movie Legends**

Supplementary Movie 1. Timelapse movie of J774A.1 macrophage cells stained with LTR phagocytosing *P. cutis* (HP28)

Supplementary Movie 2. Timelapse movie of J774A.1 macrophage cells stained with LTR phagocytosing *P. miyajii* (HP29)
